# Kinematics and workspace analysis of 4SPRR-SPR parallel robots

**DOI:** 10.1371/journal.pone.0239150

**Published:** 2021-01-20

**Authors:** Lan Luo, Li Hou, Qi Zhang, Yongqiao Wei, Yang Wu

**Affiliations:** 1 School of Mechanical Engineering, Sichuan University, Chengdu, China; 2 School of Intelligent Manufacturing, Panzhihua University, Panzhihua, China; 3 School of Mechanical & Electronical Engineering, Lanzhou University of Technology, Lanzhou, China; University of Electronic Science and Technology of China, CHINA

## Abstract

The 4SPRR-SPR parallel robot, which has considerable potential for application in the field of machining, is a novel closed-loop mechanism with a high rigid-weight ratio. Kinematics and workspace analyses of the 4SPRR-SPR parallel robot are key requirements for its application in machining. In this study, the inverse kinematics of the 4SPRR-SPR parallel robot is analyzed using a geometric method based on the mechanism arrangement of the robot. The forward kinematics model is derived by training the vector-quantified temporal associative memory (VQTAM) network, which originates from a self-organizing map (SOM). Furthermore, an improved algorithm is obtained by combining the locally linear embedding (LLE) and VQTAM methods. A boundary extraction algorithm for the workspace analysis of the parallel robot is proposed. The performance of the boundary extraction algorithm is analyzed and compared with that of a global search algorithm; the result indicates that the novel algorithm has the same computational accuracy in addition to higher efficiency. The workspace of the 4SPRR-SPR parallel robot is analyzed using the boundary extraction algorithm. Finally, the 3D model of the 4SPRR-SPR parallel robot is simulated using the ADAMS software to verify the reliability of the proposed algorithms. The simulation results demonstrate the effectiveness of the methods proposed in this study. In addition, the robot kinematics and workspace analysis methods described herein can be extended to other serial and parallel robots. This research provides a theoretical framework for trajectory planning of mechanisms, workspace optimization of robots, and robotic control.

## 1. Introduction

Parallel robots offer noticeable advantages in machining applications, especially in the field of heavy equipment manufacture, due to their high rigid-weight ratio and load capacity. However, a suitable machine tool for large-scale workpiece processing is unavailable because of the large size of the workpieces to be processed. Therefore, the processing of heavy machinery and equipment should be performed at a fixed station. Thus, it is necessary to develop processing equipment having a specific amount of flexibility and sufficient stiffness, which is capable of various forms of track processing and can bear the cutting force load. Recently, the machine tool industry has discovered the potential advantages of parallel mechanisms (PM), and many parallel machine tools have been developed based on either 6-degrees-of-freedom (DOF) or 5-DOF PMs [1]. However, fixed machine tools cannot satisfy the machining requirements in the case of a large-scale workpiece. Therefore, only mobile parallel robots can solve this problem.

Parallel robots or PMs with 5 or 6 DOFs are often used as motion simulators and crane devices. Stewart introduced a 6-DOF PM, which is now popularly known as the Stewart platform [2] and is commonly used in flight or driving simulators, vibration isolation platform, and space docking mechanisms [3]. Subsequently, lower-mobility robots (with 2–5 DOFs) have emerged and have become popular owing to their simple structure, low kinematic coupling, low cost, and convenient control [4–6]. Xie et al. [4], Huang et al. [5], and Joshi et al. [6] studied type synthesis for different types of lower-mobility PMs. Typical lower-mobility PMs such as 2-UPR-SPR PM [7] and 3-UPS-UP PM [8, 9] have been used as position adjusters (R, P, U, S stand for revolute, prismatic, universal and spherical joints, respectively). To the best of our knowledge, researchers have mainly focused on studying the type synthesis of 5-DOF PMs [5, 10–14].

The 5SPRR PM is a 6-DOF PM, which has been used as a machining tool [15–17]. The structural form of the 4SPRR-SPR parallel robot, which has great potential to transform into a new type of mobile machining robot, is derived from the 5SPRR PM. The kinematics and workspace analysis of the 4SPRR-SPR parallel robot are key factors for its application in machining. PMs have a restricted workspace compared with serial manipulators [15]. Various approaches ranging from discretization algorithms to geometrical approaches for obtaining the workspace of parallel mechanisms have been described in extensive literature [18]. Most studies on workspace analysis are mainly focused on 6-DOF PMs, especially on the Stewart platform. For instance, Fichter [19] obtained the section shape of the workspace of a 6-DOF PM based on the Stewart platform. Gosselin obtained the workspace of 6-DOF PMs through arc intersection [20]. Most researchers also analyzed the workspace of PMs using the global search algorithm proposed by Huang et al. [21, 22]; this algorithm, despite being effective and widely accepted, is inefficient.

Kinematics, which is another research focus in robotics, is of vital importance to the realization of robot control. The forward kinematics is particularly indispensable for improving the control accuracy of a robot motion controller. However, parallel manipulators do not have formulaic forward kinematic solutions. Therefore, several studies are focused on methods to simplify the mapping relationship in forward kinematics using intelligent algorithms or machine learning. Simpler mapping models of kinematics input and output are established to replace the complex analytical solution. Evolutionary computational methods such as genetic algorithm (GA) [23–27], differential evolution (DE), and particle swarm optimization (PSO) [28] are used to optimize the solutions by formulating the problem in the form of an objective or a fitness function. Furthermore, approximation methods based on artificial neural networks and polynomial regression models [29–33] can also be used to estimate forward kinematics. Robot kinematics can be regarded as a non-linear autoregressive model with exogenous input (NARX). In reference [34], the forward kinematics of parallel manipulators is solved by combining the wavelet network and the NARX method. The simplification of a complex kinematics model often leads to a decrease in the accuracy of estimation. To overcome this problem, efficient and accurate kinematics identification is needed [35]. Aquil Mirza Mohammed and Shuai Li et al. proposed a dynamic neural network for kinematic control of the Stewart platform. Furthermore, they established a model-free dual neural network to control and learn the parallel Stewart platform [36, 37]. A majority of the aforementioned research studies are focused on the forward kinematics or motion control of a typical parallel mechanism (Stewart platform), which is of great significance to other related research on parallel robots. These studies indicate that the forward kinematics solution and control of parallel robots can be realized and obtained fairly accurately using an intelligent algorithm or machine learning.

The general intelligent algorithm requires substantial iteration time and hence cannot be used for the real-time control of robots. On the contrary, machine learning can be directly used for control after the learning process, and its accuracy and real-time performance can be guaranteed. However, the validity of this method is still dependent on the hyper-parameter initialization and back-propagation learning process. Conversely, the VQTAM algorithm does not depend on the adjustment of the hyper-parameters and back-propagation learning process. It can be applied to the mapping from the input space to the output space via topology preservation, as an extension of SOM. The mathematical foundation of VQTAM is nonlinear manifold embedding. This method is suitable for time series modeling and prediction of nonlinear systems. Depending on the SOM structure, the discrete mapping relationship can effectively realize output estimation according to exogenous input [35, 38, 39]. Since the input space and output space of VQTAM are discrete, a network with more neurons is required to achieve high-precision prediction, which decreases the efficiency. An improved local linear algorithm of VQTAM is proposed based on LLE to improve the computational accuracy without decreasing the efficiency.

This paper describes a novel parallel robot that could be used as a mobile machining robot and proposes the forward kinematics of the 4SPRR-SPR parallel robots based on a VQTAM neural network. In addition, a new boundary extraction algorithm for workspace analysis is developed. On the one hand, a VQTAM neural network can improve the efficiency of an algorithm in a practical control application and ensure real-time control compared to kinematics based on an intelligent algorithm or machine learning. On the other hand, it can avoid the influence of super parameters of machine learning algorithms on learning efficiency and accuracy. The forward kinematics based on VQTAM is not only suitable for a 4SPRR-SPR parallel robot but also can be extended to the kinematics of other parallel and serial robots. The boundary extraction algorithm improves the efficiency of workspace analysis and can also be applied to different types of robots. The main chapters of the article are arranged as follows: Section 2 outlines the architecture and general kinematic properties of the 4SPRR-SPR parallel robots; the inverse kinematics of the 4SPRR-SPR robots is studied in Section 3; Section 4 describes the nonlinear system identification method based on VQTAM, obtains the forward kinematics of parallel robot, and establishes the training method of VQTAM neural network; in Section 5, a general and efficient algorithm for workspace analysis of PMs is presented, and the workspace analysis of 4SPRR-SPR parallel robots is performed with this algorithm; in Section 6, a simulation based on the 3D model of the 4SPRR-SPR parallel robot is performed to verify the reliability and effectiveness of the proposed algorithms.

## 2. 4SPRR-SPR parallel robots

**Fig 1:**
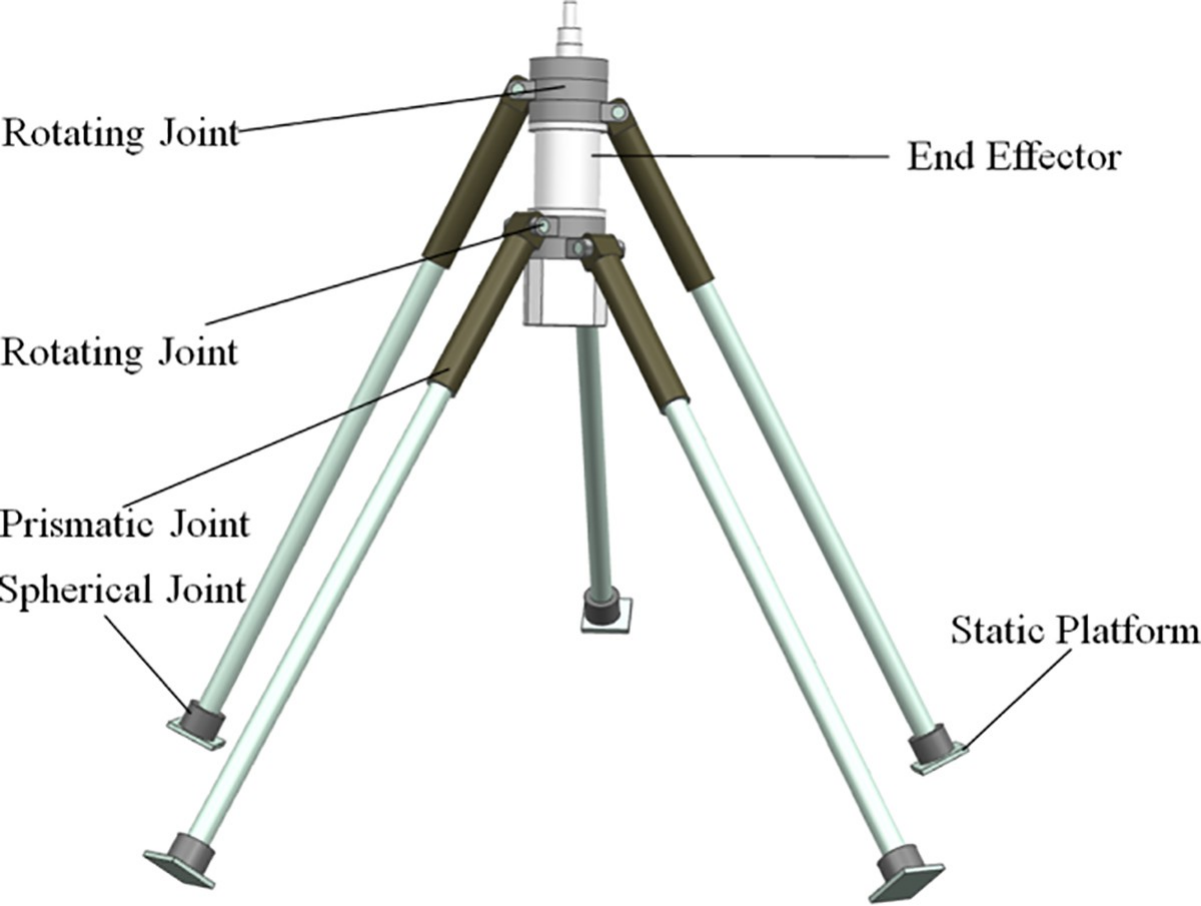
The structure of a 4SPRR-SPR parallel robot.

The 4SPRR-SPR parallel robot is one of the new 5-DOF PMs, and its structure is shown in Fig 1. The four limbs of the PM consist of one spherical joint, a moving pair, and two rotating pairs, and the other one lack for a rotating pair at one end of the limb. The rotating pairs connected with an end effector are coaxial. The spherical joints connected with the static platform are not arranged in the same plane but are distributed in different positions in space. This provides more possibilities for the optimization of the parallel mechanism.

**Fig 2:**
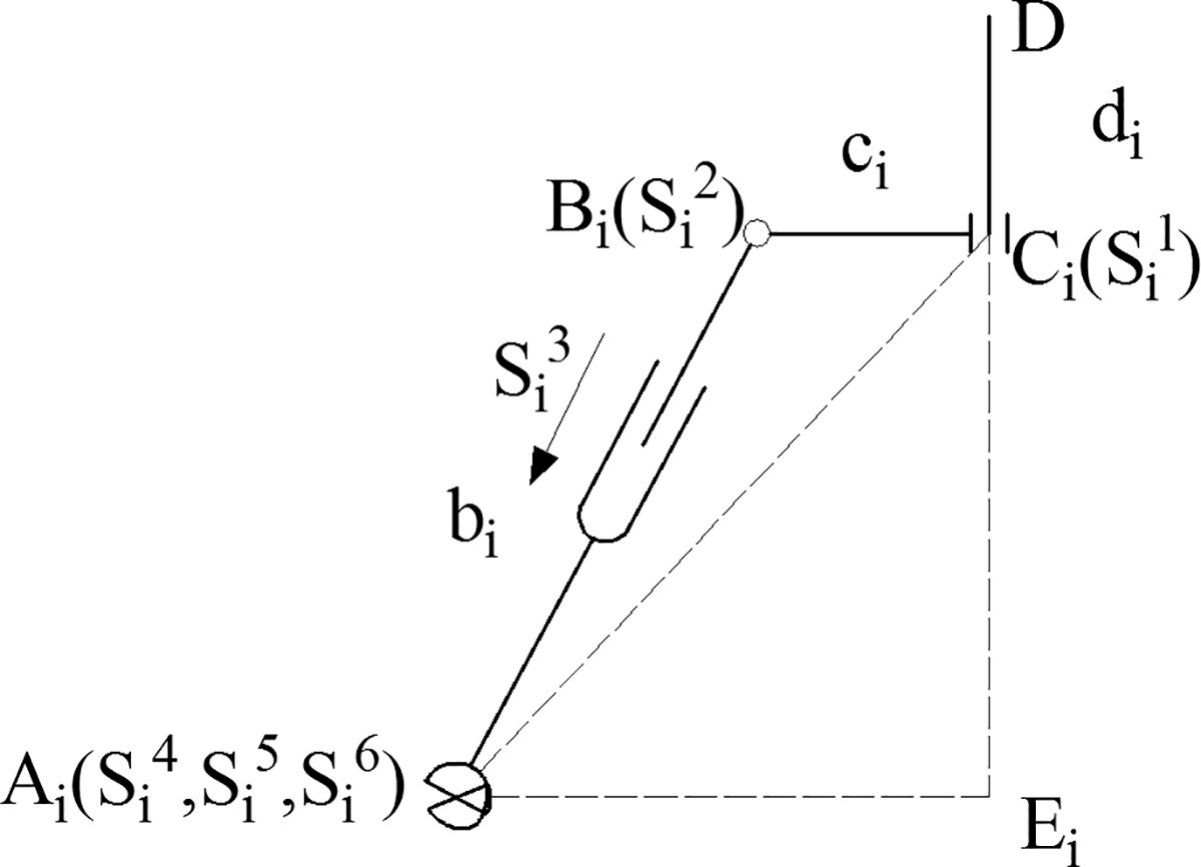
*i*th limb structure diagram.

The arrangements of the kinematic pairs of the 4SPRR-SPR parallel robot are similar, except that the end of the first limb is fixed to the moving platform. The kinematic pair arrangement of the *i*^th^ chain is shown in Fig 2 (*i* = 2, 3,…, 5).

*A*_*i*_ is the center point of the universal joint connected by the *i*th limb and the static platform, *B*_*i*_ and *C*_*i*_ are the two revolving pair centers of the limb, and *D* is the reference point of the position of the end effector. The screw of the centerline ***S*** of each pair is used to represent the motion of the pair [20]. ***S***_*i*_^1^ and ***S***_*i*_^2^ are revolting joints; ***S***_*i*_^3^ is a prismatic joint; and ***S***_*i*_^4^, ***S***_*i*_^5^, and ***S***_*i*_^6^ constitute the rotation of the spherical joint in three directions.

For the *i*th limb, the coordinate system *O*_*i*_*-x*_*i*_*y*_*i*_*z*_*i*_ at *E*_*i*_ is established. *E*_*i*_ is the vertical foot from *A*_*i*_ to *DC*_*i*_. The screws represented in coordinated *O*_*i*_*-x*_*i*_*y*_*i*_*z*_*i*_ are

$$\left\{ \begin{array}{l} S_{i}^{1}=(0,0,1;0,0,0)\\ S_{i}^{2}=(0,1,0;m_{i},0,c_{i})\\ S_{i}^{3}=(0,0,0;n_{i},0,m_{i})\\ S_{i}^{4}=(0,1,0;0,0,n_{i})\\ S_{i}^{5}=(1,0,0;0,0,0)\\ S_{i}^{6}=(0,0,1;0,-n_{i},0) \end{array}\right.,$$ (1)

where *b*_*i*_, *c*_*i*_, *m*_*i*_, and *n*_*i*_ represent the length of segments *A*_*i*_*B*_*i*_, *B*_*i*_*C*_*i*_, *C*_*i*_*E*_*i*_, and *E*_*i*_*A*_*i*_, respectively.

The six screws of the *i*^th^ limb (*i* = 2, 3,…, 5) are linearly independent. The screws of the *i*^th^ limb constitute a 6-system of screws, which provides no constraint to the end effector.

The screw system of the first limb differs from those of the others because the end of the first limb is fixed to the moving platform. The screws represented in coordinated *O*_*1*_*-x*_*1*_*y*_*1*_*z*_*1*_ are

$$\left\{ \begin{array}{l} S_{1}^{2}=(0,1,0;m_{1},0,c_{1})\\ S_{1}^{3}=(0,0,0;n_{1},0,m_{1})\\ S_{1}^{4}=(0,1,0;0,0,n_{1})\\ S_{1}^{5}=(1,0,0;0,0,0)\\ S_{1}^{6}=(0,0,1;0,-n_{1},0) \end{array}\right..$$ (2)

The reciprocal screw of the 5-system screws can be written as

$$S_{1}^{r}=(0,1,0;0,0,n_{1}).$$ (3)

Therefore, five limbs provide one constraint to the end effector. It can be confirmed that the 4SPRR-SPR parallel robots have 5 DOFs. The motion screw system, which reflects the DOF property of the end-effector, can be obtained by finding the secondary inverse screw system of the end-effector.

$$\begin{array}{l} S_{1}^{pm}=S_{1}^{rr}=(1,0,0;0,0,0)\\ S_{2}^{pm}=S_{2}^{rr}=(0,1,0;0,0,0)\\ S_{3}^{pm}=S_{3}^{rr}=(0,0,0;1,0,0)\\ S_{4}^{pm}=S_{4}^{rr}=(0,0,0;0,0,1)\\ S_{5}^{pm}=S_{5}^{rr}=(0,0,1;0,-n_{1},0) \end{array}.$$ (4)

Eq (4) shows that the end effector can rotate around the X and Y axes and around the line (0,0,1;0,-*n*_1_,0), which passes parallel to the Z-axis through point *A*_1_. The end effector can also move along the X and Z axes.

## 3. Inverse kinematics of a 4SPRR-SPR parallel robot

The prismatic joints of the five limbs constitute the drive of a 4SPRR-SPR parallel robot. Therefore, the inverse position solution of the parallel robot can be regarded as the calculation of the rods *A*_*i*_*B*_*i*_ about the length *l*_*i*_ using the coordinates of the known points *D* and direction vectors ***S***^1^ (*i* = 1, 2, …, 5). The directions of the end effector are the same for the five limbs in unified coordinates.

The geometric condition of the parallel robot is determined using Eqs (1) and (2).

$$\left\{ \begin{array}{l} c_{i}⊥S^{1}\\ S_{i}^{2}⊥c_{i}\\ b_{i}⊥S_{i}^{2}\\ S^{1}⊥S_{i}^{2} \end{array}\right.,$$ (5)

where ***b**_i_* = ***A**_i_**B**_i_*, ***c**_i_* = ***B**_i_**C**_i_*, and ***d**_i_* = ***C**_i_**D**_i_*.

As shown in Fig 2, the *i*^th^ limb is considered for the analysis. *E*_*i*_ is the vertical foot from *A*_*i*_ to *DC*_*i*_. From the geometric relation, the result is deduced using Eqs (6)–(8).

$$E_{i}C_{i}=(A_{i}C_{i}\cdot S^{1})S^{1}.$$ (6)

$$A_{i}E_{i}=A_{i}C_{i}-E_{i}C_{i}.$$ (7)

$$c_{i}=c_{i}(\frac{A_{i}E_{i}}{\left|A_{i}E_{i}\right|}).$$ (8)

The coordinates of points *C*_*i*_ and *B*_*i*_ can be obtained upon calculating *c*_*i*_. Accordingly, the input parameter *l*_*i*_ corresponding to the pose can be obtained.

The main structural parameters of 4SPRR-SPR parallel robots are the coordinates of points *A*_*i*_ (*i* = 1, 2,…, 5) and rod lengths *c*_*i*_ and *d*_*i*_. The above parameters can be used to determine the corresponding inverse kinematics calculation of the parallel robot.

The inverse kinematics solution of the robot is calculated considering the structural parameters shown in Table 1 as an example.

**Table 1:** The structural parameters of the parallel robot.

Parameters	Values (mm)
*A*_1_	(0,2380.19, 1823.98)
*A*_2_	(-790.54, 1011.96, 1877.15)
*A*_*3*_	(790.54,1011.96, 1877.15)
*A*_*4*_	(-557.90, 2471.63, 2218.87)
*A*_*5*_	(557.90, 2471.63, 2218.87)
*d*_*1*_	440
*d*_*2*_	520
*d*_*3*_	600
*d*_*4*_	1040
*d*_*5*_	1120
*c*_*i*_	180

By determining the position and posture of the moving platform, the coordinate of point D and the direction vector ***S***^1^ is found to be (0,1119.17,3641.27) and (0,0,-1), respectively. The length *l*_*i*_ of the rod *A*_*i*_*B*_*i*_, as seen in Table 2, is calculated by substituting these parameters in the aforementioned algorithm.

**Table 2:** The calculated rod length *l*_*i*_ values of the parallel robot.

The rod length label	The rod length(mm)
*l*_1_	1751.26
*l*_2_	1386.05
*l*_3_	1317.89
*l*_4_	1338.79
*l*_5_	1318.17

**Fig 3:**
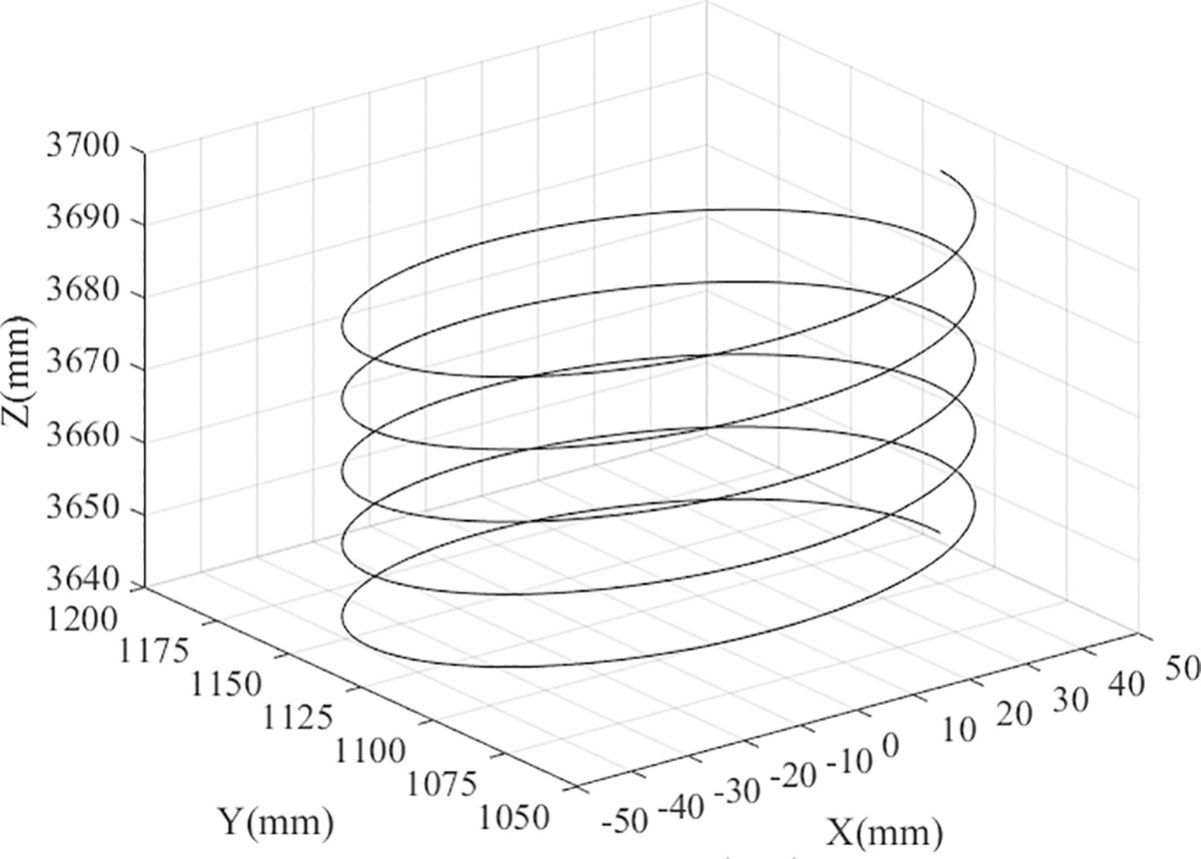
The reference point trajectory of the end effector.

The inverse solutions of the poses can be obtained by discretizing the trajectory of the end effector. Additionally, the velocity and acceleration of the moving pair and the corresponding length of the bar can be obtained. Considering the machining and using the parallel robot as an example, the reference point trajectory of the moving platform is observed to be a helix, and the direction vector is (0, 0, -1). The reference point trajectory of the moving platform is shown in Fig 3.

According to the discrete pose of the end effector, the rod length corresponding to each point can be inversely solved, as shown in Table 3.

**Table 3:** Inverse solution results for different positions.

Coordinates of reference points (mm)	*l*_1_(mm)	*l*_2_(mm)	*l*_3_(mm)	*l*_4_(mm)	*l*_5_(mm)
(50,1119.17,3641.27)	1751.87	1411.80	1295.41	1357.77	1300.34
(49.98,1120.74,3641.32)	1750.94	1411.93	1295.57	1356.40	1298.93
(49.90,1122.30,3641.37)	1750.01	1412.03	1295.75	1355.01	1297.54
(49.78,1123.87,3641.42)	1749.09	1412.12	1295.95	1353.60	1296.17
**…**	**…**	**…**	**…**	**…**	**…**

**Fig 4:**
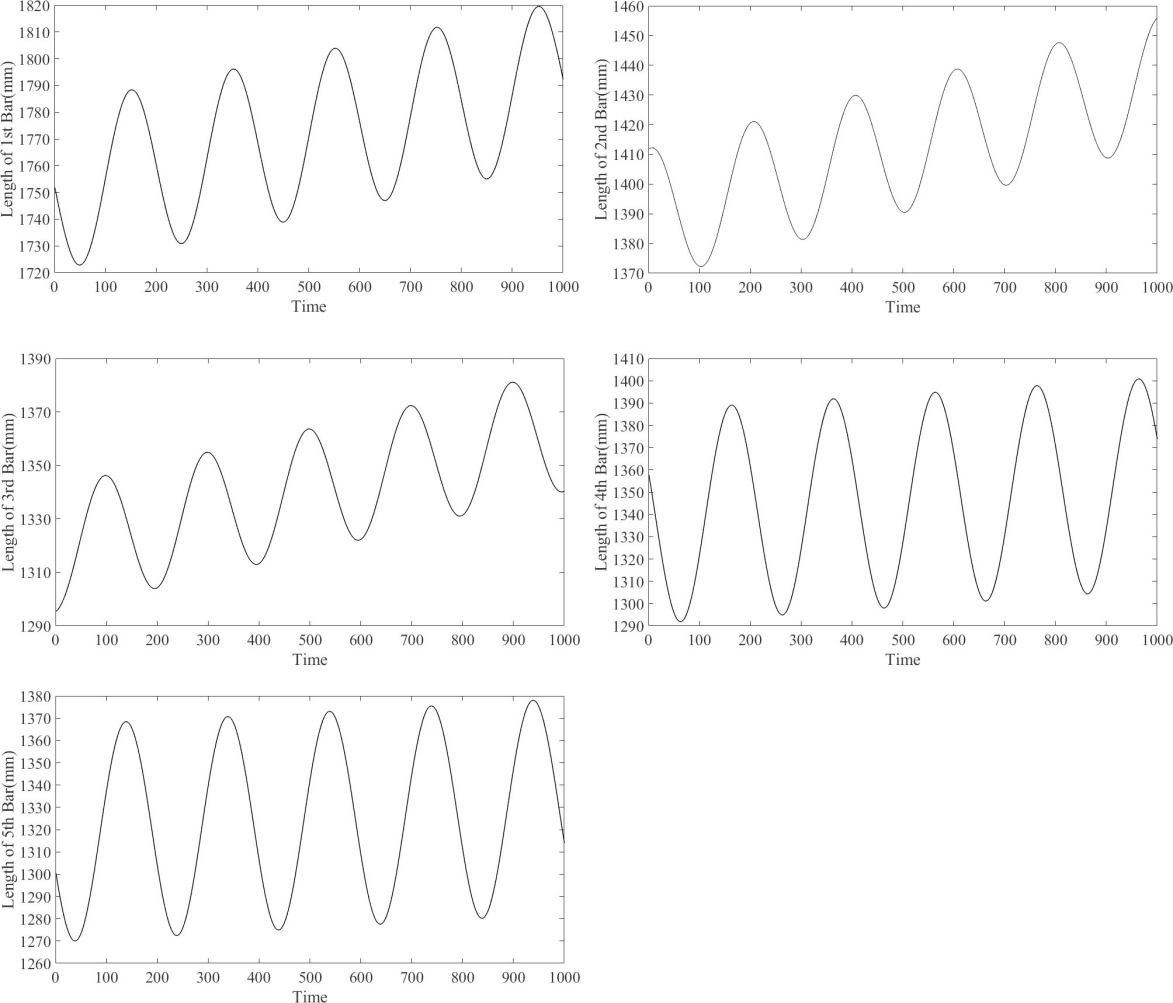
Plots for variation in rod lengths for different positions during motion of end effector. (A) The length of 1st rod. (B) The length of 2nd rod. (C) The length of 3rd rod. (D) The length of 4th rod. (E) The length of 5th rod.

The variation in the rod lengths can be plotted according to the inverse solution results for different positions during the motion of the end effector, as shown in Fig 4A–4E.

The length of each rod varies during the processing of workpiece holes on the moving platform (Fig 4). The horizontal axis in Fig 4 represents the time of motion, and the iteration steps in the calculation process are considered as a unit time. The specific motion time can be determined based on the actual working conditions.

**Fig 5:**
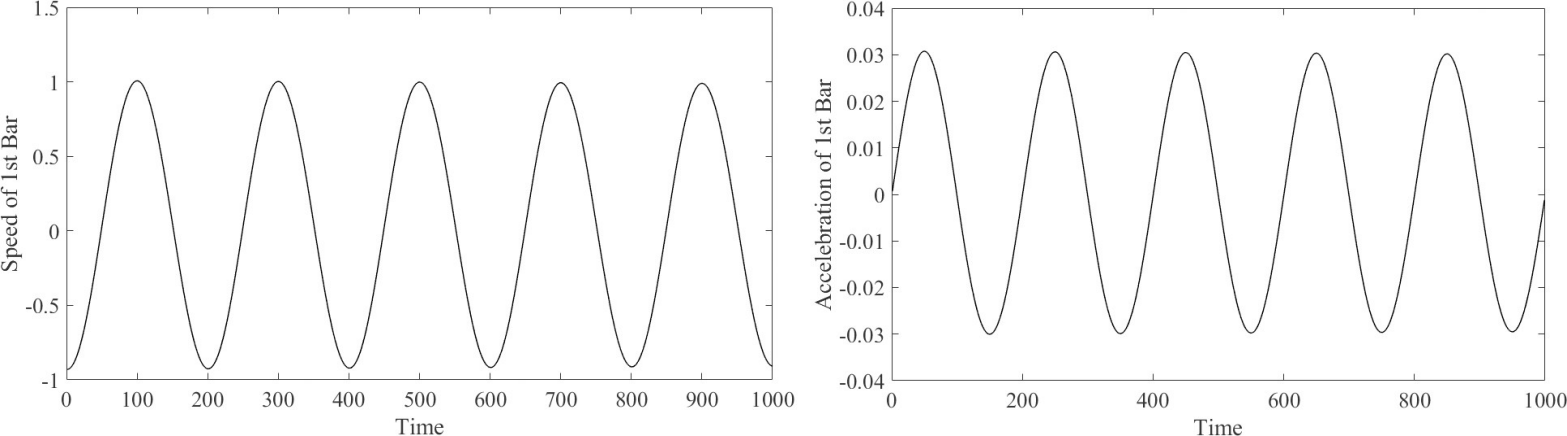
The speed and acceleration of 1^st^ rod. (A) The speed of 1^st^ rod. (B) The acceleration of 1^st^ rod.

The length of the motion can be differentiated to obtain the velocity, which can be differentiated again to obtain the acceleration. In this example, the velocity and acceleration of each rod are obtained using the difference between the lengths of each rod, according to the iteration step. Fig 5 shows the speed and acceleration considering rod 1 as an example.

The velocity and acceleration obtained are regarded as dimensionless data because no definite time is used in the calculation. The numerical value in Fig 5 only reflects the trend of change. In practical application, the time parameters can be determined according to the actual conditions of the maximum torque and speed of the motor, to realize the control of the motion of the end effector.

The inverse kinematic solutions determine the motion relationship between the output and input of a 4SPRR-SPR parallel robot, which is a theoretical basis for mechanism control. Furthermore, it provides algorithm support for subsequent workspace analysis.

## 4. Forward kinematics of a 4SPRR-SPR parallel robot

The forward kinematics of the parallel robot aims at solving the corresponding pose given the length of drive limbs. The pose ***Q*** of the end effector and the length ***p*** of the drive limbs are regarded as output and input, respectively. The forward kinematics of the robot can be transformed into nonlinear dynamic system identification.

### 4.1 System identification of nonlinear dynamical system using VQTAM

The discrete-time difference formula is established as shown in Eq (9) [35, 39].

$$Q(t+1)=f[Q(t),\ldots ,Q(t-n_{q}+1);p(t),\ldots ,p(t-n_{u}+1)],$$ (9)

where ***Q***(*t*) = [*x*(*t*),*y*(*t*),*z*(*t*);***S***^1^(*t*)], which is the pose of the end effector; the length of the drive limb at time *t* is ***p***(*t*) = [*l*_1_(*t*),*l*_2_(*t*),*l*_3_(*t*);*l*_4_(*t*),*l*_5_(*t*)]. *f*(∙) is the mapping function of the system. The output ***Q*** at *t*+1 is determined using the previous *n*_*q*_ outputs and *n*_*p*_ inputs.

The purpose of forward kinematics is to recognize the mapping function *f*(∙) from input to output. For a non-linear problem without formulaic solutions, simplifying the model can guarantee efficiency and ensure favorable real-time performance in engineering applications.

VQTAM can be effectively used as an extension of SOM to realize non-linear system identification. Nonlinear manifold embedding is used in VQTAM to establish nonlinear mapping. The feature of SOM is the simulation of competition and cooperation among neurons. Different neurons are activated for different inputs in the mapping process, and the output can be obtained from the combination of the activated neurons. There are three layers in the VQTAM neural network: input space ***ω***^in^, output space ***ω***^out^, and lattice space. ***X***^*in*^ and ***X***^*out*^ can be expressed as shown in Eqs (10) and (11).

$$X^{in}=[Q(t),\ldots ,Q(t-n_{q}+1);p(t),\ldots ,p(t-n_{u}+1)].$$ (10)

$$X^{out}=[Q(t+1)].$$ (11)

The time-series data can be constructed as training samples of VQTAM. The training samples are used to build a topological structure of a neural network embedded in a multi-dimensional data space. Thus, the mapping relationship from input to output can be constructed.

The input and output space are composed of the weight vectors ***ω***_*i*_^in^ and ***ω***_*i*_^out^, respectively, where *i* is the index of a neuron, which reflects the topological location in lattice space. There is a consistent one-to-one match between the weight vectors and neurons in the lattice space. The dimensions of weight vectors ***ω***_*i*_^in^ and ***ω***_*i*_^out^ are the same as corresponding input vectors ***X***^*in*^ and ***X***^*out*^, respectively. An activated neuron corresponds to the nearest weight vector ***ω***_i*_^in^ to input ***X***^in^. i* is the index of activated neurons, as shown in Eq (12), where *A* is the collection of all neuron indexes in the lattice space [39].

$$i*=\underset{i\in A}{\arg\;\min}\{\left\|X^{in}-\omega_{i}^{in}\right\|\}.$$ (12)

***ω***_i*_^out^ is obtained using the neuron index i*, and the estimation of output *X*^*out*^ is shown in Eq (13).

$${\hat{X}}^{out}=\omega_{i*}^{out}.$$ (13)

The learning process of VQTAM mainly involves searching the i* of activated neurons and updating the weight vectors ***ω***_i*_^in^ and ***ω***_i*_^out^. The weight vectors ***ω***_*i*_^in^ and ***ω***_*i*_^out^ of activated neurons and their neighborhoods are updated in this process.

The influence range parameters *σ*(*t*) of the activated neuron and the learning speed *α*(*t*) decrease exponentially with the learning epoch to improve the convergence rate in the learning process, as shown in Eqs (14) and (15).

$$\alpha (t)=\alpha_{0}{\left(\alpha_{T}/\alpha_{0}\right)}^{t/T}.$$ (14)

$$\sigma (t)=\sigma_{0}{\left(\sigma_{T}/\sigma_{0}\right)}^{t/T},$$ (15)

where *t* is the current learning epoch, and *T* is the total learning epoch. *α*_0_ and *σ*_0_ are the initial values, and *α*_*T*_ and *σ*_*T*_ are the parameter values of training epoch *T*.

The Gauss neighborhood function is used to determine the effect of input on the neighbors of the activated neuron, as shown in Eq (16).

$$h(i*,i;t)=\exp(\frac{-{\left\|i(t)-i*(t)\right\|}^{2}}{\sigma^{2}(t)}).$$ (16)

***ω***_*i*_^in^ and ***ω***_*i*_^out^ are updated as shown in Eqs (17) and (18).

$$\omega_{i}^{in}⇐\alpha (t)h(i*,i;t)[X^{in}-\omega_{i}^{in}].$$ (17)

$$\omega_{i}^{out}⇐\alpha (t)h(i*,i;t)[X^{out}-\omega_{i}^{out}].$$ (18)

To sum up, the steps involved in the VQTAM algorithm are as follows:

**VQTAM algorithm**:

Begin

(training part)

1 Input: ***X***^*in*^ and ***X***^*out*^ in training set

2 Search for an activated neuron according to ***X***^*in*^

$$i*=\underset{i\in A}{\arg\;\min}\{\left\|X^{in}-\omega_{i}^{in}\right\|\}$$

3 Update the weight vectors of the neuron

$$\omega_{i}^{in}⇐\alpha (t)h(i*,i;t)[X^{in}-\omega_{i}^{in}]$$

$$\omega_{i}^{out}⇐\alpha (t)h(i*,i;t)[X^{out}-\omega_{i}^{out}]$$

4 Continue execution until termination conditions are met.

(testing part)

5 Input: ***X***_*test*_^*in*^ in testing set

6 Search for an activated neuron according to ***X***_*test*_^*in*^

$$i*=\underset{i\in A}{\arg\;\min}\{\left\|X_{test}^{in}-\omega_{i}^{in}\right\|\}$$

7 Output:

$${\hat{X}}_{test}^{out}=\omega_{i*}^{out}$$

### 4.2 VQTAM LLE algorithms

The VQTAM algorithm is used to quantify the input space ***ω***^in^ and the output space ***ω***^out^. The neurons in ***ω***^in^ and ***ω***^out^ correspond through mapping relations. The input ***X***^*in*^ is approximated to the nearest neuron ***ω***_i*_^in^ during estimation. The accuracy of the estimation results can be guaranteed when the number of neurons is adequate. However, an increase in the number of neurons increases the network size and decreases the computing efficiency. Thus, local linearization of the activated node is used, which can balance the number of neurons and improve the prediction accuracy. An improved locally linear embedding (LLE) algorithm based on local linearization for VQTAM is proposed. The inverse kinematics of the robot can be further optimized, which can ensure computational efficiency.

Local linearization is performed in VQTAM networks. The search algorithm is used to search the nearest *n* data points ***ω***_i*n_^in^ in the input space, and the corresponding output data ***ω***_i*n_^out^ are mapped.

It is assumed that input data can be represented by a linear combination of several samples in its neighborhood. That is, the input *X*^*in*^ to be predicted is represented by a linear combination of *n* data points in its neighborhood in the input space ***ω***_i*n_^in^.

$$X^{in}=\sum_{k=1}^{n}c_{k}\omega_{i*k}^{in}.$$ (19)

The output has the same linear combination.

$${\hat{X}}^{out}=\sum_{k=1}^{n}c_{k}\omega_{i*k}^{out},$$ (20)

where *c*_*k*_ is the coefficient of the linear combination. The output can be estimated using Eq (20) after solving *c*_*k*_. The cost function is defined as shown in Eq (21) and is rewritten in the form of a matrix.

$$\begin{array}{l} J(c)={\left\|X^{in}-\sum_{k=1}^{n}c_{k}\omega_{i*k}^{in}\right\|}^{2}\\ \;={\left\|X^{in}-Yc\right\|}^{2} \end{array}.$$ (21)

The least-squares problem can be solved using singular value decomposition (SVD). ***Y*** can be decomposed as shown in Eq (22).

$$\begin{array}{l} SVD(Y)=[U][T][V^{T}]\\ \;where\;Y\in {\mathbb{R}}^{m\times m},U\in {\mathbb{R}}^{m\times l},V\in {\mathbb{R}}^{l\times l} \end{array}.$$ (22)

The column vectors of ***U*** are the left singular vectors of ***Y***. The column vectors of ***V***^*T*^ are the right singular vectors of ***Y***.

$$T=\left[\begin{array}{c} \Sigma \\ 0 \end{array}\right],$$ (23)

Where ***Σ*** is a diagonal matrix whose value is the singular value *υ* of matrix ***Y***, and ***U*** and ***V***^*T*^ are orthogonal matrices.

***U*** can be disassembled as [***U***_*n*_,***U***_*m-n*_]. Eq (24) shows the solution obtained using SVD for the least-squares problem given by Eq (21).

$$c=V\Sigma^{-1}U_{n}^{T}X^{in}.$$ (24)

### 4.3 Forward kinematics of 4SPRR-SPR parallel robot based on VQTAM

The poses for the sample set are generated according to the robot parameters listed in Table 1, using Eqs (25)–(30). The generated data can effectively cover the entire workspace of the manipulator.

$$x(t)=1500(1-e^{-\pi t})\cos1.88\pi t,$$ (25)

$$y(t)=1500(1-e^{-\pi t})\sin1.88\pi t+1500,$$ (26)

z(t)=2000cost+2000, (27)

$$\theta_{1}(t)=\pi \sin t,$$ (28)

$$\theta_{2}(t)=2\pi (1-e^{-\pi t})\sin0.86\pi t,$$ (29)

$$S^{1}=\left[\begin{array}{ccc} \cos\theta_{1}\sin\theta_{2} & \sin\theta_{1}\sin\theta_{2} & \cos\theta_{2} \end{array}\right],$$ (30)

where *t*∈[0,1000] and step *t* = 0.1. Thus, ***Q***(*t*) = [*x*(*t*),*y*(*t*),*z*(*t*);***S***^1^(*t*)] is calculated. The three components of ***S***^1^ are *s*_*x*_, *s*_*y*_ and *s*_*z*_, respectively. ***p***(*t*) = [*l*_1_(*t*),*l*_2_(*t*),*l*_3_(*t*);*l*_4_(*t*),*l*_5_(*t*)] is calculated using the inverse kinematics algorithm. ***p***(*t*) and ***Q***(*t*) are used as the sample set. Thus, the VQTAM network is trained. The data for training and testing are selected randomly in each epoch.

Table 4 shows the hyperparameter setting of the VQTAM network training. A VQTAM network of dimension 80×80 (*M*_*x*_×*M*_*y*_) is trained based on the hyperparameters setting, and the effect of training is evaluated using the test set.

**Table 4:** Hyperparameter setting of VQTAM.

*n*_*q*_	*n*_*p*_	*epoch*	*α*_*0*_	*α*_*M*_	*σ*_*0*_	*σ*_*M*_
3	3	5000	0.8	0.001	15	0.001

Root mean squared error (RMSE), R squared (R^2^), and root mean absolute error (RMAE) are used to evaluate the prediction accuracy of VQTAM for forward kinematics. Table 5 shows the prediction accuracy of the six parameters in output calculated using RMSE, R^2^, and RMAE.

**Table 5:** RMSE, R^2^, and RMAE of standard VQTAM network.

	*x*	*y*	*z*	*s*_*x*_	*s*_*y*_	*s*_*z*_
RMSE	0.0544	0.0542	0.0689	0.0861	0.0792	0.1425
R^2^	0.9758	0.9768	0.9600	0.9725	0.9519	0.9649
RMAE	0.7230	0.8522	1.5152	0.7455	1.7239	1.1008

**Fig 6:**
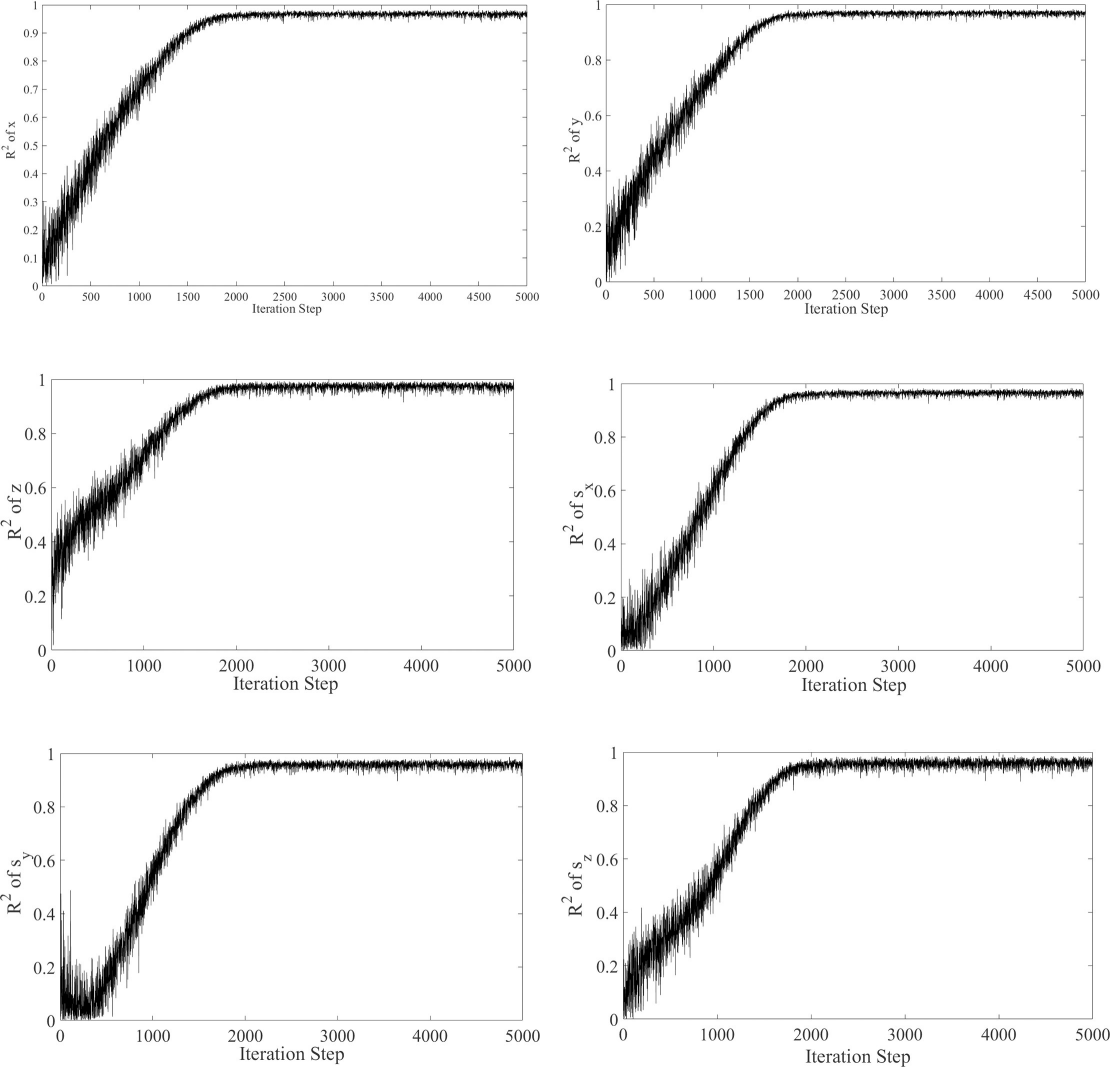
Convergence result. (A) Prediction accuracy of x in the training process. (B) Prediction accuracy of y in the training process. (C) Prediction accuracy of z in the training process. (D) Prediction accuracy of *s*_*x*_ in the training process. (E) Prediction accuracy of *s*_*y*_ in the training process. (F) Prediction accuracy of *s*_*z*_ in the training process.

The value of R^2^ is between 0 and 1. The closer it is to 1, the better is the effect of the regression fitting, which means that the learning VQTAM network is more accurate as an approximate model. Generally, R^2^ > 0.95 can be used in engineering applications. For VQTAM networks of dimension 80×80, the R^2^ values after learning are all above 0.95. This shows that this network is suitable for engineering applications. The RMSE and RMAE reflect the prediction errors in the VQTAM network, and their small values also indicate the prediction accuracy of the network. The convergence result in the neural network training process is shown in Fig 6. It can be seen that the R^2^ of the VQTAM neural network tested using the test set in the training process gradually increases and converges to approximately 1. This confirms the effectiveness of the VQTAM neural network training algorithm.

**Fig 7:**
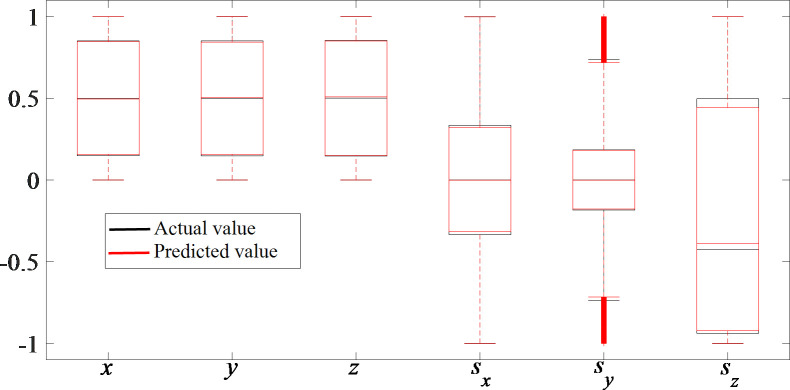
VQTAM network prediction effect box diagram.

The consistency between the actual data and the estimated data can be displayed more clearly through box diagrams, as shown in Fig 7. The box diagrams of the actual values and predicted values are notably similar, which verifies the excellent prediction ability of the VQTAM network with dimensions of 80×80.

**Fig 8:**
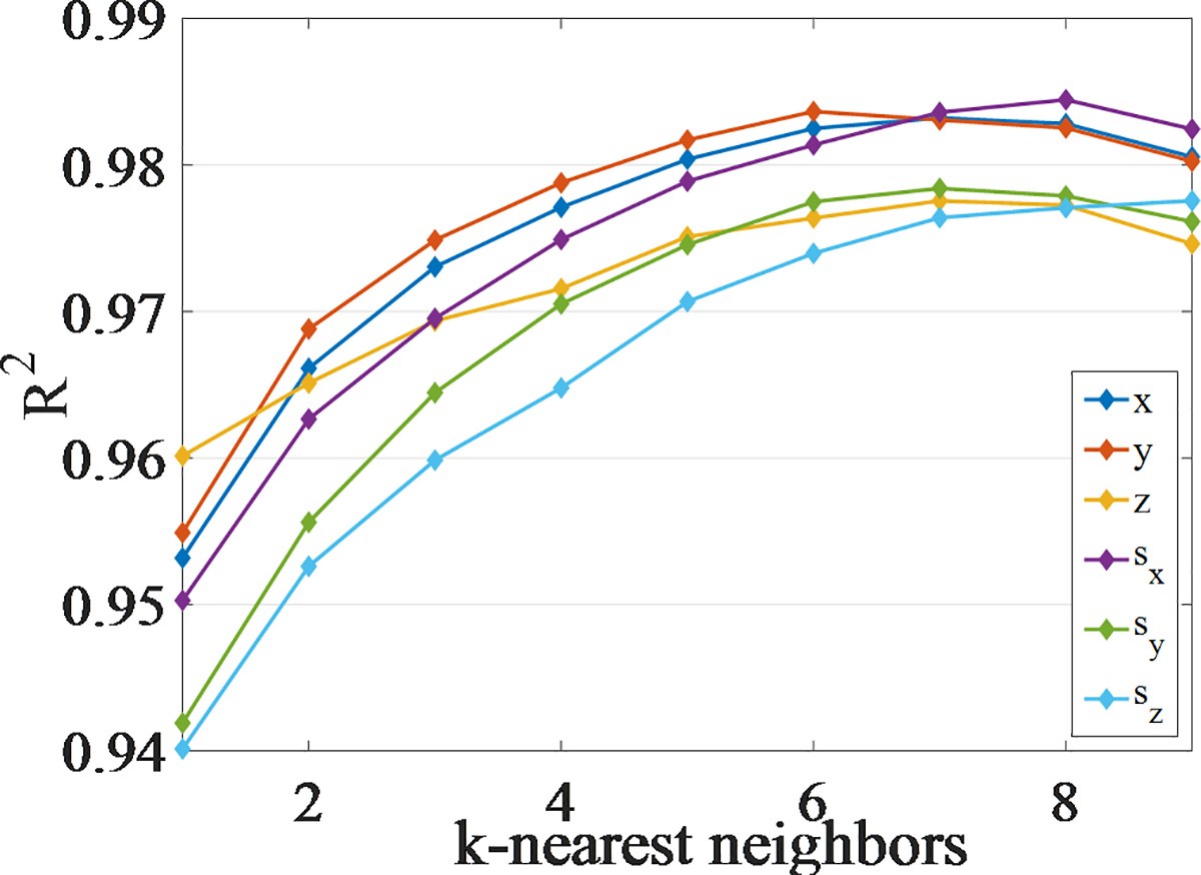
The influence of the k-nearest neighbor number in the improvement algorithm of VQTAM with LLE.

The output of the inverse kinematics problem is estimated using the VQTAM local linear improvement algorithms. The priority search K-means tree algorithm is used to search the neighbor data of the input. The influence of parameter *k* on the prediction accuracy is analyzed considering a VQTAM network of dimension 70×70 as an example, as shown in Fig 8.

**Fig 9:**
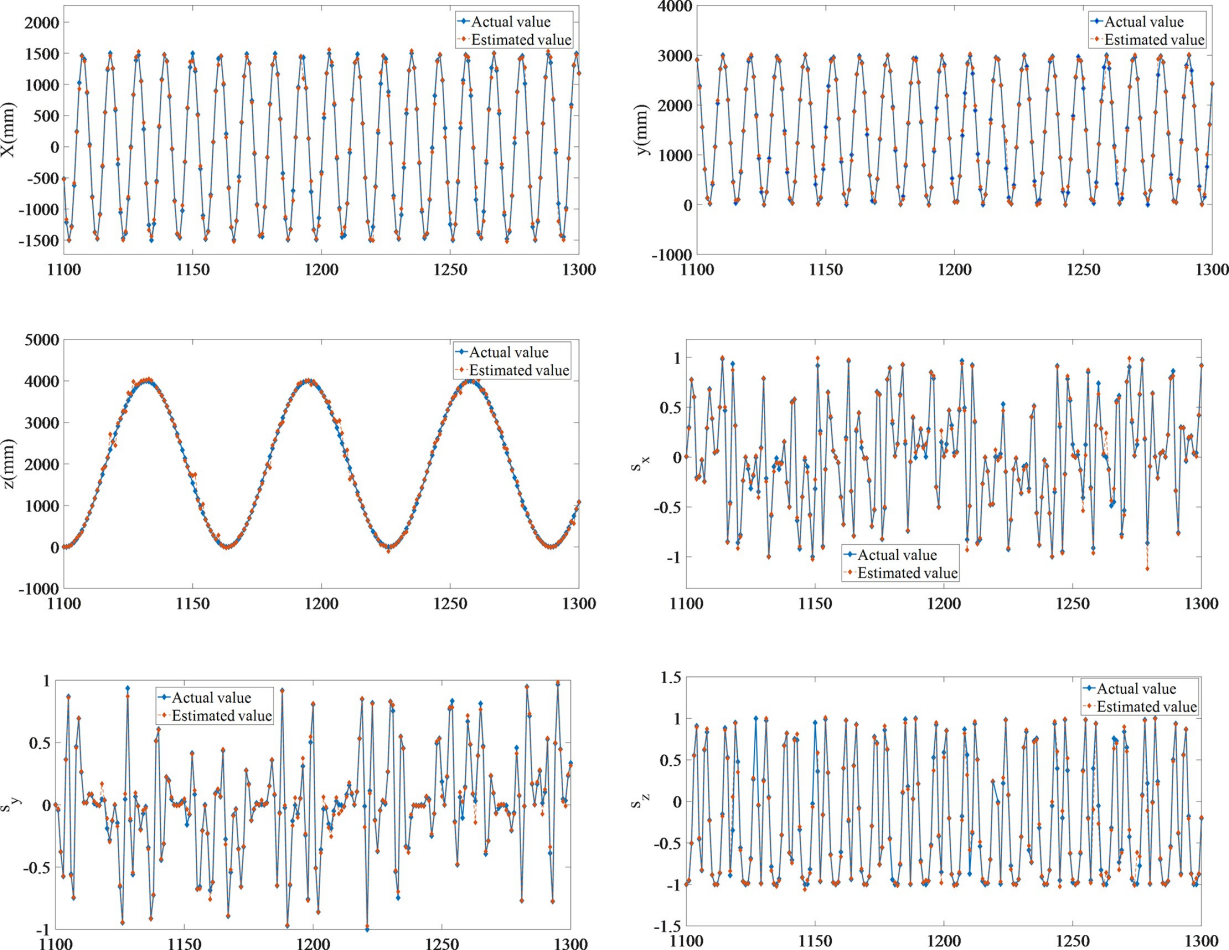
Estimation of forward kinematics output. (A) Estimation of forward kinematics output of *x*. (B) Estimation of forward kinematics output of *y*. (C) Estimation of forward kinematics output of *z*. (D) Estimation of forward kinematics output of *s*_*x*_. (E) Estimation of forward kinematics output of *s*_*y*_. (F) Estimation of forward kinematics output of *s*_*z*_.

Fig 8 shows that an increase in the number of neurons results in a significant increase in the prediction accuracy of the four algorithms. Local linearization of VQTAM yields remarkable results. Using the improved VQTAM algorithm, the prediction accuracy of a network of dimension 50×50 is found to be close to that of the standard VQTAM network of dimension 70×70. The output of the forward kinematics and the poses of the end effector are estimated as shown in Fig 9. The estimated poses are compared with the actual poses. This shows that the VQTAM neural network can effectively estimate the output.

The error of VQTAM can be obtained by comparing the result with the actual pose of the robot. The RSME for position estimation is less than 0.1 mm, as shown in Table 6. This precision has met the control requirements of general robots in industrial applications.

**Table 6:** The RSME of the estimated inverse kinematics output.

Parameter	*x*(mm)	*y*(mm)	*z*(mm)
RSME	0.0352	0.0327	0.0418
Parameter	*s*_*x*_	*s*_*y*_	*s*_*z*_
RSME	0.0494	0.0443	0.0918

## 5. Workspace boundary extraction algorithm

Workspace refers to the space that the actuator can reach, and its size is an important parameter for measuring the performance of the mechanism. The working space of a parallel robot is limited by the length of the link, the rotation angle of the rotating pair, and the interference condition of the connecting rod.

The premise of workspace analysis is the forward or inverse position solutions of the robot. Currently, workspace analysis is solved as a global search problem. The general solution approach is to determine the possible spatial range (larger than and containing the workspace) of the mechanism and to judge whether the points within the spatial range meet the constraints according to certain step size. This algorithm is computationally complex, consumes substantial computing resources, and is therefore inefficient.

The workspace boundary must be a closed and continuous surface. After determining the boundary point, there must be other boundary points in its neighborhood. By iteratively searching the neighborhood of the boundary points, all points on the boundary of the workspace can be determined, and the workspace analysis can be completed.

### 5.1 Boundary point determinate algorithm

Based on the definition of topological space, the workspace *X* of a mechanism can be regarded as a subspace of 3-D Euclidean space. For set *X*, there exist points *a* that satisfy ***a***∈*X*, and points in *X* and *X*' exist in any neighborhood *U* of *a*.

$$U\cap X\neq \emptyset \&U\cap X^{\prime }\neq \emptyset .$$ (31)

Therefore, *a* is defined as the boundary of set *X*.

a∈∂(X). (32)

Subsequently, the boundary of set *X* can be calculated as shown in Eq (33), where *X*° is the interior of set *X*, which is constituted by the interior point of *X*, and *X*′ is the complement of set *X*.

$$\partial (X)=X^{-}\cap {X^{\prime }}^{-}=(X{}^{\circ}\cup X^{\prime }{}^{\circ}{)}^{\prime }.$$ (33)

The relation between these two is as follows:

X°=X'−'. (34)

According to the aforementioned relationship, the concept of neighborhood is extended in 3D space coordinates based on the numerical algorithm, and the step size is set as h.

**Fig 10:**
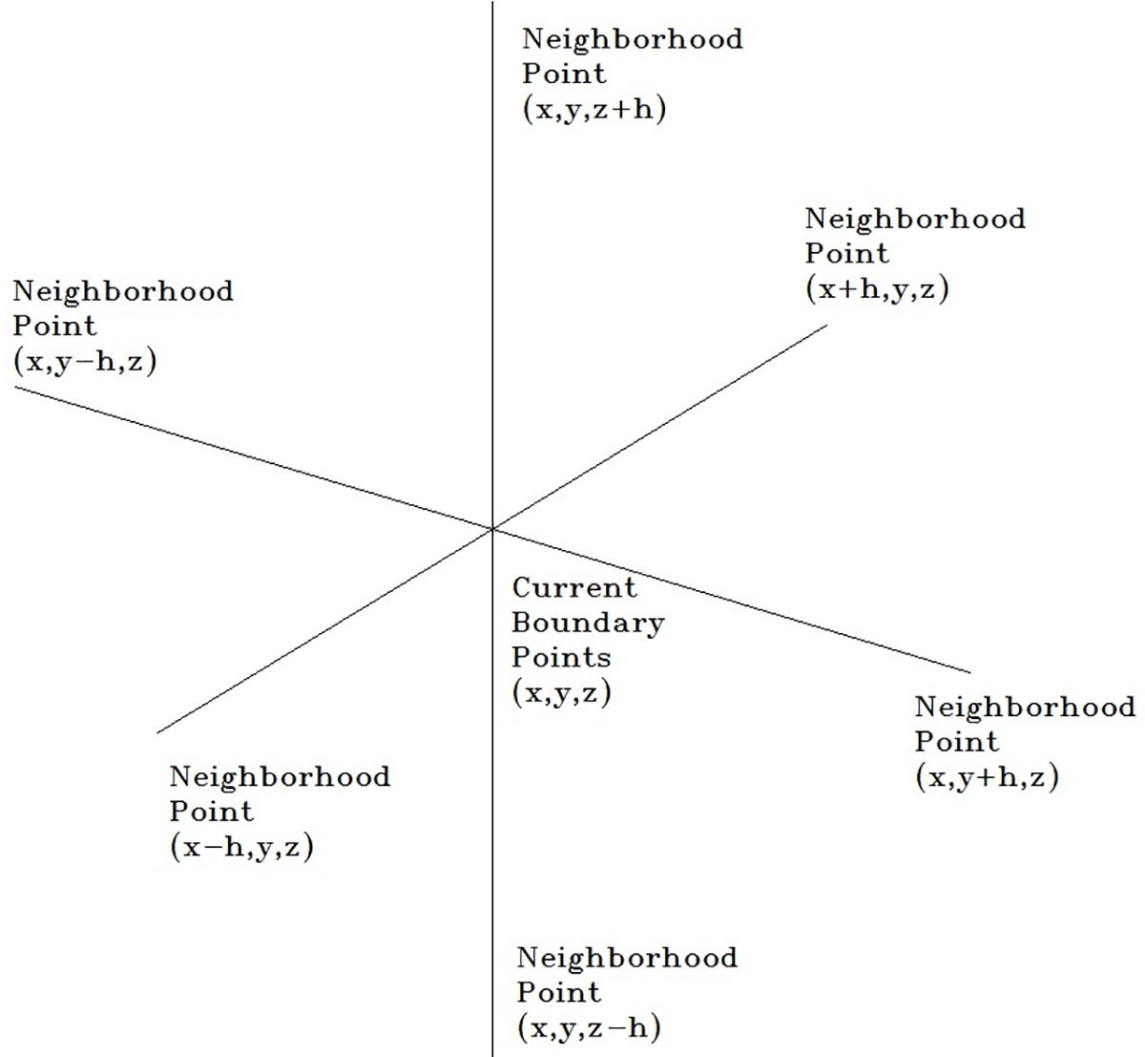
Point and its neighborhood points in space.

Assuming *a* = (*x*,*y*,*z*), the points in its neighborhood *U* are (*x+h*,*y*,*z*), (*x-h*,*y*,*z*), (*x*,*y+h*,*z*), (*x*,*y-h*,*z*),(*x*,*y*,*z+h*), and (*x*,*y*,*z-h*), respectively, for *u*_*i*_ (*I* = 1, 2, …, 6). Fig 10 shows the neighbors of a point in 3-D Euclidean space ${\mathbb{R}}^{3}$.

**Fig 11:**
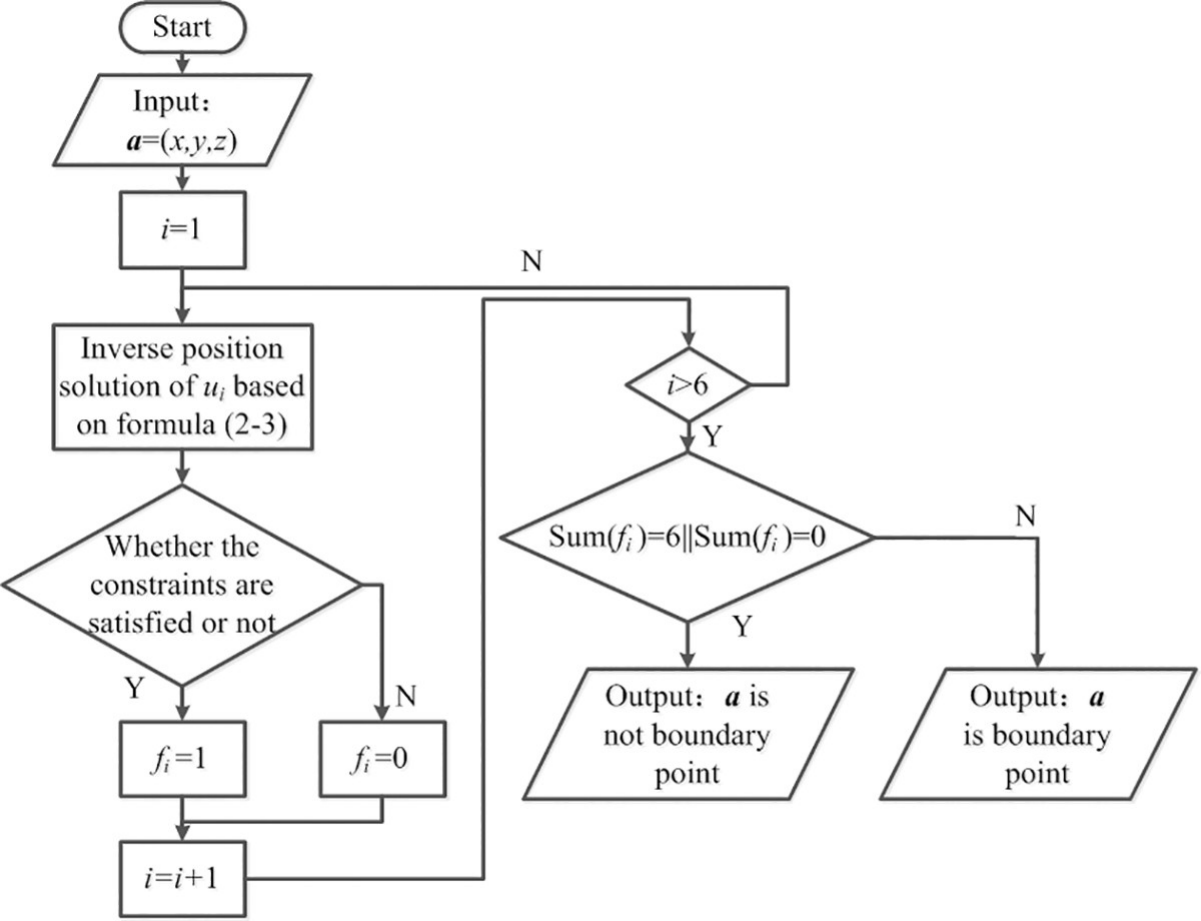
Boundary point determinate algorithms.

In the space where the robot is located, when one part of the point in the neighborhood satisfies the constraint condition and the other part does not, the point can be determined as the boundary point of the workspace. Fig 11 shows the algorithm for determining workspace boundary points according to Eqs (32) and (33).

### 5.2 Boundary point search algorithms

The boundary for set *X* is a closed and continuous surface in space, which is defined as shown in Eq (35).

f(x,y,z)=0. (35)

Therefore, the workspace can be expressed as a closed space area, as shown in Eq (36).

X={(x,y,z)|f(x,y,z)≤0}. (36)

Surface *f*(*x*, *y*, *z*) = 0 is a continuous closed surface in space. Let *P*_0_(*x*_0_, *y*_0_, *z*_0_) be a point on the boundary of *X*, i.e. ∀ε>0, ∃δ>0. When (*x*, *y*, *z*) = *U*(*P*_0_, *δ*),

$$\left|f(x,y,z)-f(x_{0},y_{0},z_{0})\right|<\varepsilon .$$ (37)

Specifically, there must be other boundary points in the neighborhood of the boundary point *P*_0_(*x*_0_, *y*_0_, *z*_0_). In the numerical algorithm, if the allowable range is reasonably determined, the remaining boundary points can be searched in the range of step size.

**Fig 12:**
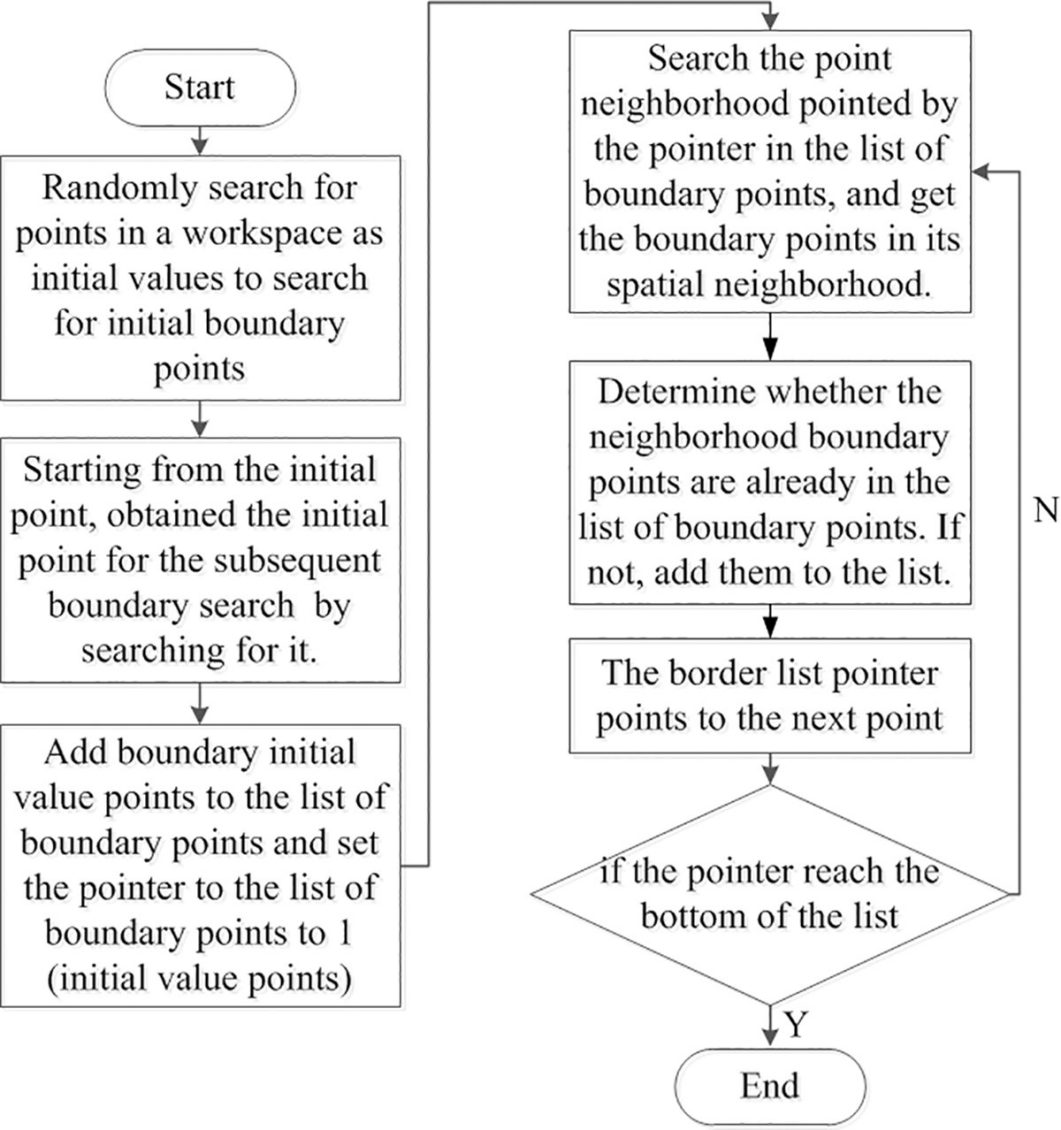
Workspace analysis algorithms.

Thus, a boundary point search algorithm can be established. Fig 12 shows the flow chart of the algorithm. First, it is necessary to determine a boundary point as the initial point of iterative search of boundary points. Thereafter, the surface can be reached by advancing along any direction from the inside of the surface because the workspace of the mechanism is a closed surface. The boundary points can be obtained as the initial iteration points by starting from a point in the workspace and searching in a certain direction.

The initial boundary point is added to the boundary point list, and the corresponding pointer to the boundary list is set as 1 (depending on the programming environment) to ensure that it points to the initial point. The neighborhood of the point, which is pointed by the pointer of the boundary point list, is searched, and it is judged whether the neighborhood boundary points have been found in the boundary point list. The new boundary points are added to the boundary point list. The boundary list pointer increments itself by 1, and the aforementioned operation is repeated. The pointer reaching the bottom of the list signifies the end of the loop. To observe the convergence of the algorithm, the distance between the pointer and the bottom of the list can be used as the criterion for convergence during the calculation. For the algorithm to converge, the number of points on the closed surface must be limited when the step size is fixed.

### 5.3 Algorithm analysis

The functional relationship between the time increment order and the input scale of the two algorithms, which is expressed by the asymptotic symbol *O*, is established to compare the time complexities of the boundary extraction algorithm and global search algorithm. In the search process of the two algorithms, it is necessary to judge whether the point satisfies the constraints. The time complexities of the processes are the same; hence, the time cost is set as 1. For a certain region to be searched, the input size should be negatively correlated with the search step size and positively correlated with the search step number. The time complexity of the global search algorithm can be represented by *O*(*N*^3^) if the search steps in all three directions are *N*. The global search algorithm can be executed in the Cartesian, cylindrical, or spherical coordinate systems, but the choice of the coordinate system does not affect its time complexity.

In the boundary extraction algorithm, it is necessary to judge whether the current point is a boundary point or not. In the worst-case scenario, it is necessary to judge whether the six points in its neighborhood satisfy the constraints. The time complexity of the process is 6, which pertains to the constant level. The coefficient can be neglected in the description of the time growth order.

The entire process of boundary point search involves traversing the points on the workspace surface. Thus, the search scale is positively correlated with the workspace surface area. In summary, the time complexity of the boundary search algorithm should be *O*(*N*^2^). Therefore, the time increment of the boundary extraction algorithm is lower than that of the global search algorithm. In the case of large-scale input, the efficiency of the boundary search algorithm is evidently better than that of the global search algorithm.

Taking the Stewart robot as an example, the traditional global search algorithm and boundary search algorithm are applied to analyze the workspace, and the two algorithms are compared.

**Fig 13:**
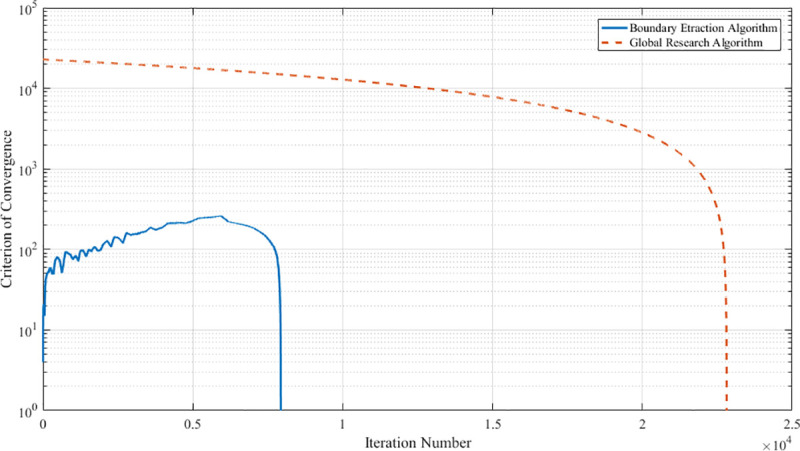
Convergence curves of algorithms.

As shown in Fig 13, the convergence curve of the boundary extraction algorithm is below that of the global search algorithm. It illustrates that the boundary extraction algorithm is computationally less complex and more efficient.

**Fig 14:**
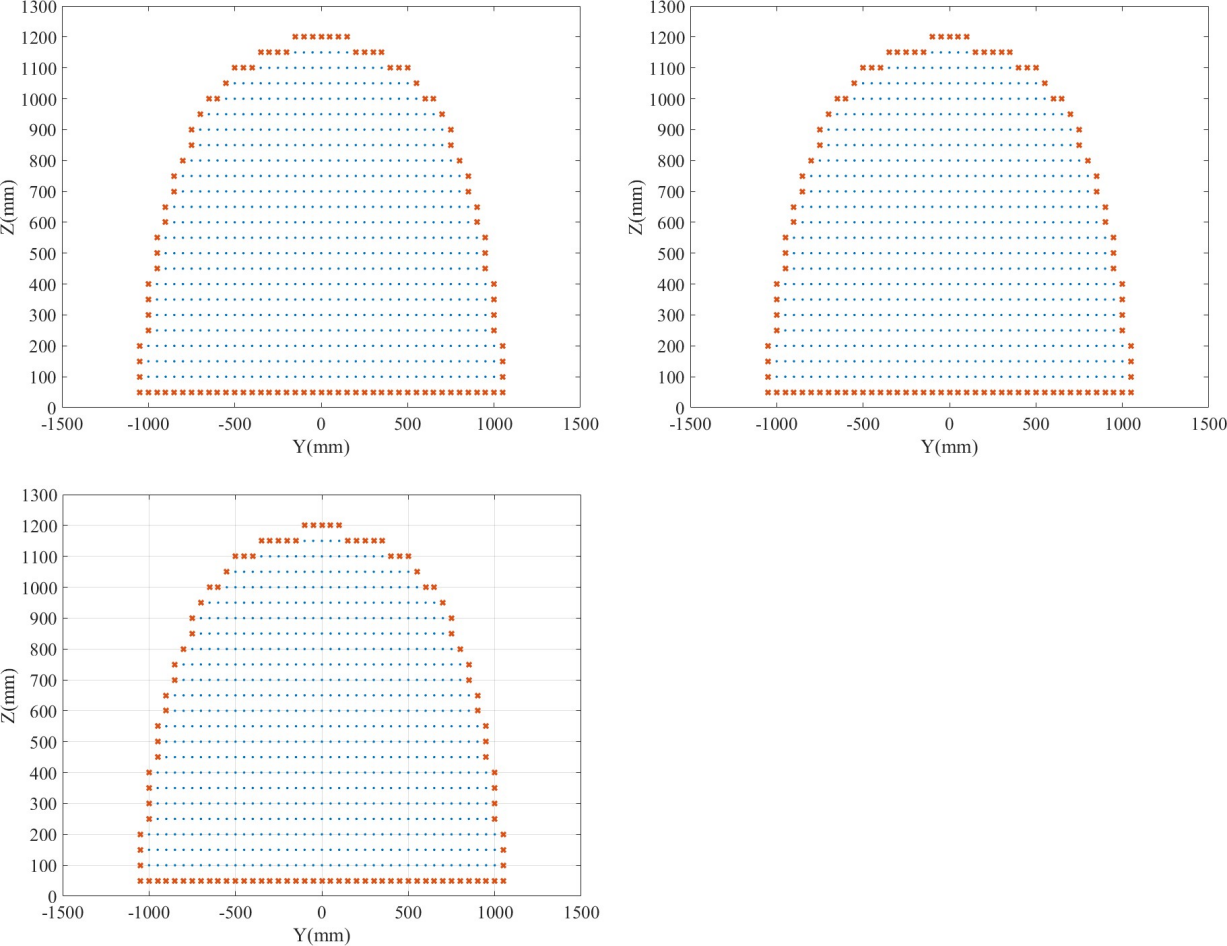
Computed results of algorithms. (A) X = 0. (B) X = 50. (C) X = -50.

Fig 14 shows the computed results on different sections of the two algorithms. There is no difference between the two results in the representation of the workspace. Therefore, the accuracy of the boundary extraction algorithm is the same as that of the global search algorithm.

In summary, the boundary extraction algorithm has the same computational accuracy but a higher efficiency compared to the global search algorithm.

### 5.4 Rods length conditions and rotating joints angle conditions

The workspace analysis of the parallel robot can be completed by determining the workspace boundary after obtaining the constraint conditions, such as the rod length, the rotation angle of the kinematic pair, and the interference condition of the connecting rod.

The driving rod lengths corresponding to different positions and postures of the mechanism's moving platform can be calculated through the inverse kinematics of the 4SPRR-SPR parallel mechanism. However, the variation range of the rod length is limited. When the rod length reaches its limit, the reference point on the moving platform reaches the boundary of the workspace. Using *l*_*max*_ and *l*_*min*_ to represent the maximum and minimum values of the *i*^th^ rod, respectively, the constraints on the length of the rods are shown in Eq (38).

$$l_{\min}\leq l_{i}\leq l_{\max}.$$ (38)

Because various types of rotating joints are involved (generalized rotating joints, including rotating joints, spherical joints, etc.), the determination of the constraints for the rotating pairs of the 4SPRR-SPR parallel mechanism is more complicated. The rotating joints ***S***^1^ connecting the rods and the end effector of this robot have no restriction for the rotation angle and can rotate around its axis. On the other hand, the rotating joints ***S***_*i*_^2^ have a restriction for the rotation angle and must avoid interference between the connecting rod and the moving platform. According to the robot arrangement characteristics, the angle between the connecting rod ***c***_*i*_ and the axis of the end effector is constant at 90°. Taking the direction of the connecting rod as the datum of the rotating pair and denoting the turning angle of the rotating pair as *θ*_*i*_, the conditions for avoiding the interference between the connecting rod and the moving platform are shown in Eq (39).

$$-90{}^{\circ}\leq \theta_{i}\leq 90{}^{\circ}.$$ (39)

The calculation of angle *θ* is given by Eq (40).

$$\theta_{i}=\arccos\frac{b_{i}\cdot c_{i}}{\left|b_{i}\cdot c_{i}\right|}.$$ (40)

Spherical joints are rotating joints with 3 DOFs. Eq (41) shows the constraint condition for 4SPRR-SPR parallel robots.

$$\theta_{Pi}=\arccos\frac{b_{i}\cdot n_{Pi}}{\left|b_{i}\right|}\leq \theta_{P\max},$$ (41)

where ***n***_*Pi*_ denotes the attitude of the *i*^th^ spherical joint sub-base in the coordinate system.

**Fig 15:**
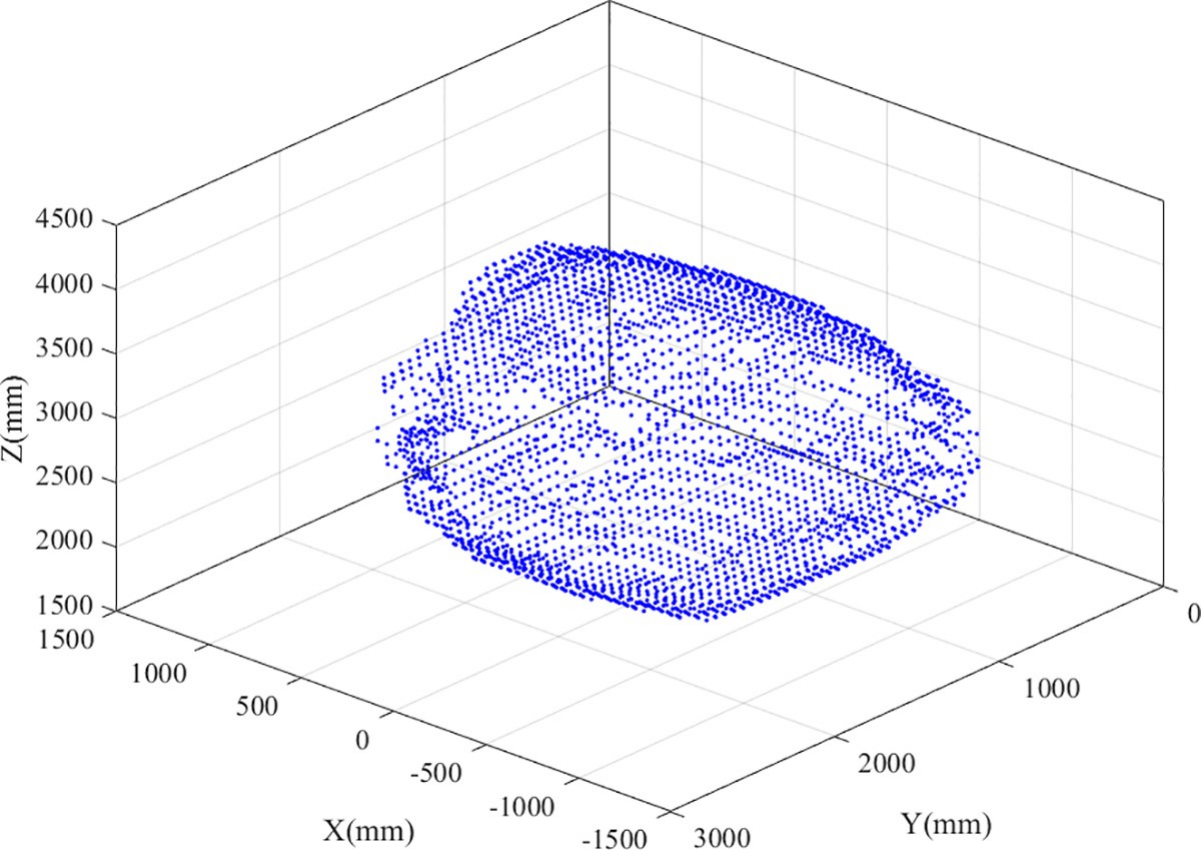
Workspace boundary points.

The workspace of the parallel robot is analyzed using the boundary point search algorithm, considering the parameters listed in Table 1 as an example. Furthermore, the boundary points of the workspace are obtained by searching, as shown in Fig 15.

**Fig 16:**
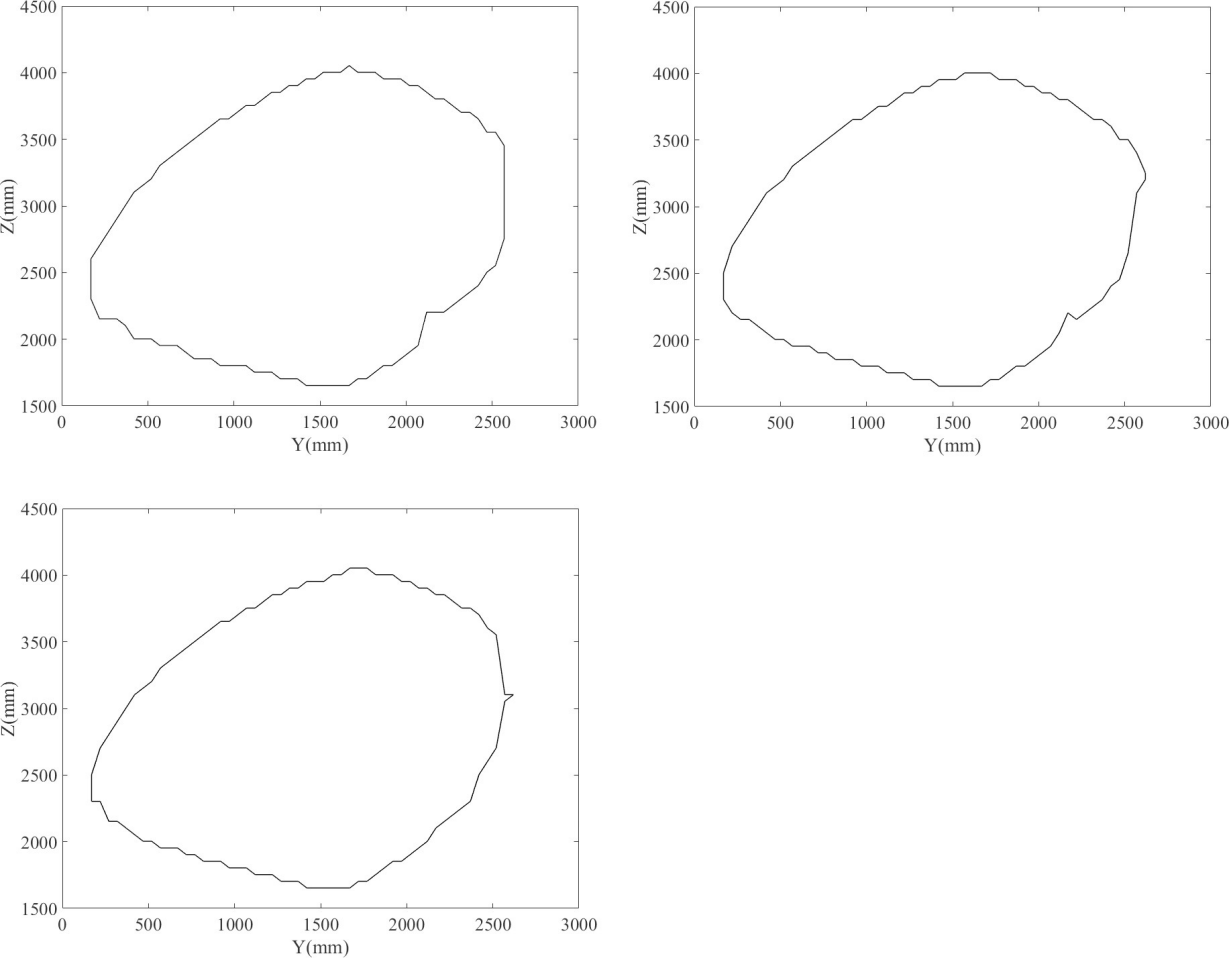
Workspace boundaries for different sections. (A) X = -100. (B) X = 0. (C) X = 100.

The boundary points, after being processed, on different sections are connected using the convex hull algorithm. The results are shown in Fig 16.

**Fig 17:**
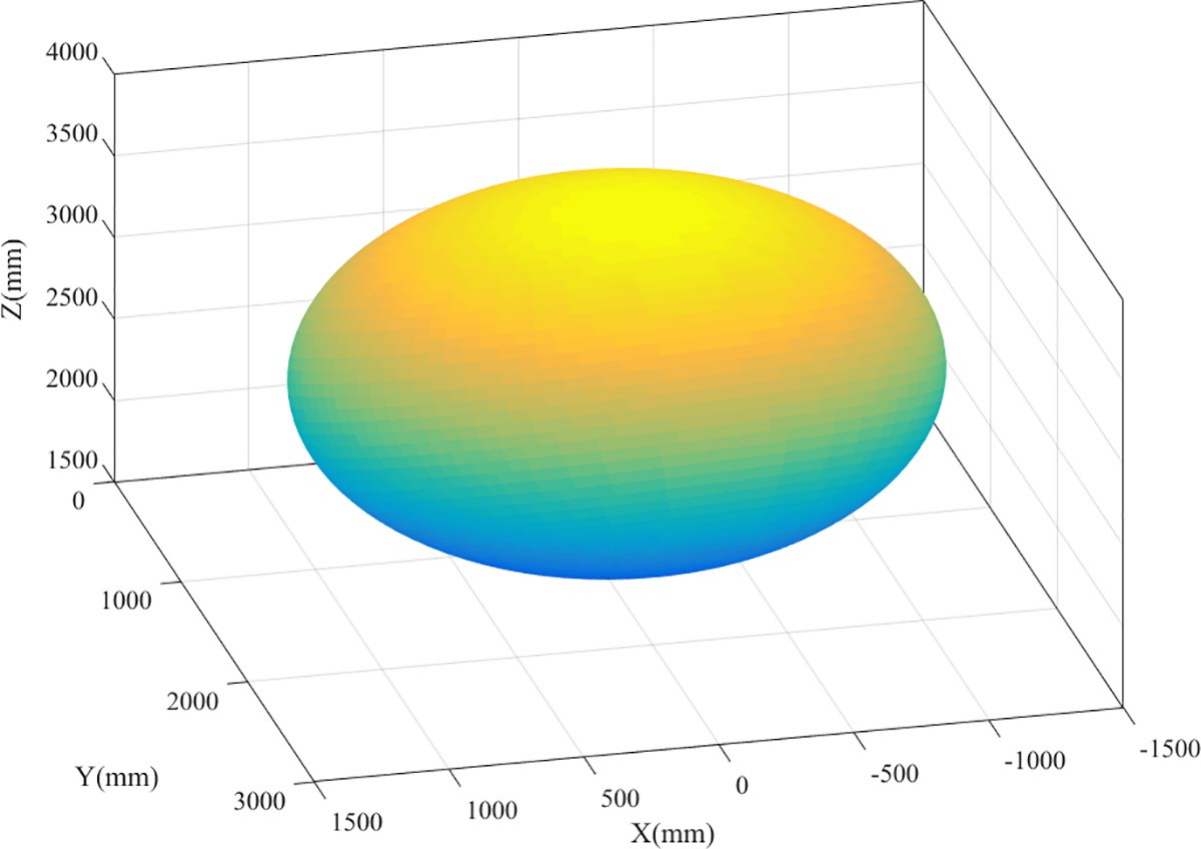
Boundary surface fitting.

In practical applications, the boundary point of the workspace is determined, which is convenient for mathematical description, fitting, and simplification of the workspace. As shown in Fig 17, the workspace boundary points of the parallel robot are obtained using the boundary search algorithm, and then the workspace boundary is fitted to an oblique ellipsoid in space using the least-squares method. In practical engineering applications and subsequent optimization analysis, the workspace of the parallel mechanism can be regarded as an ellipsoid, without any significant impact on the workspace volume calculation and the position and attitude accessibility of the end effector. Using the parameters in Table 1 as an example, the volume around the boundary points, the volume of the ellipsoid after fitting, and the relative error are calculated using numerical integration to be 5.9141×10^9^ mm^3^, 6.4170×10^9^ *mm*^3^, and 7.84%, respectively.

## 6. Simulation and discussion

**Fig 18:**
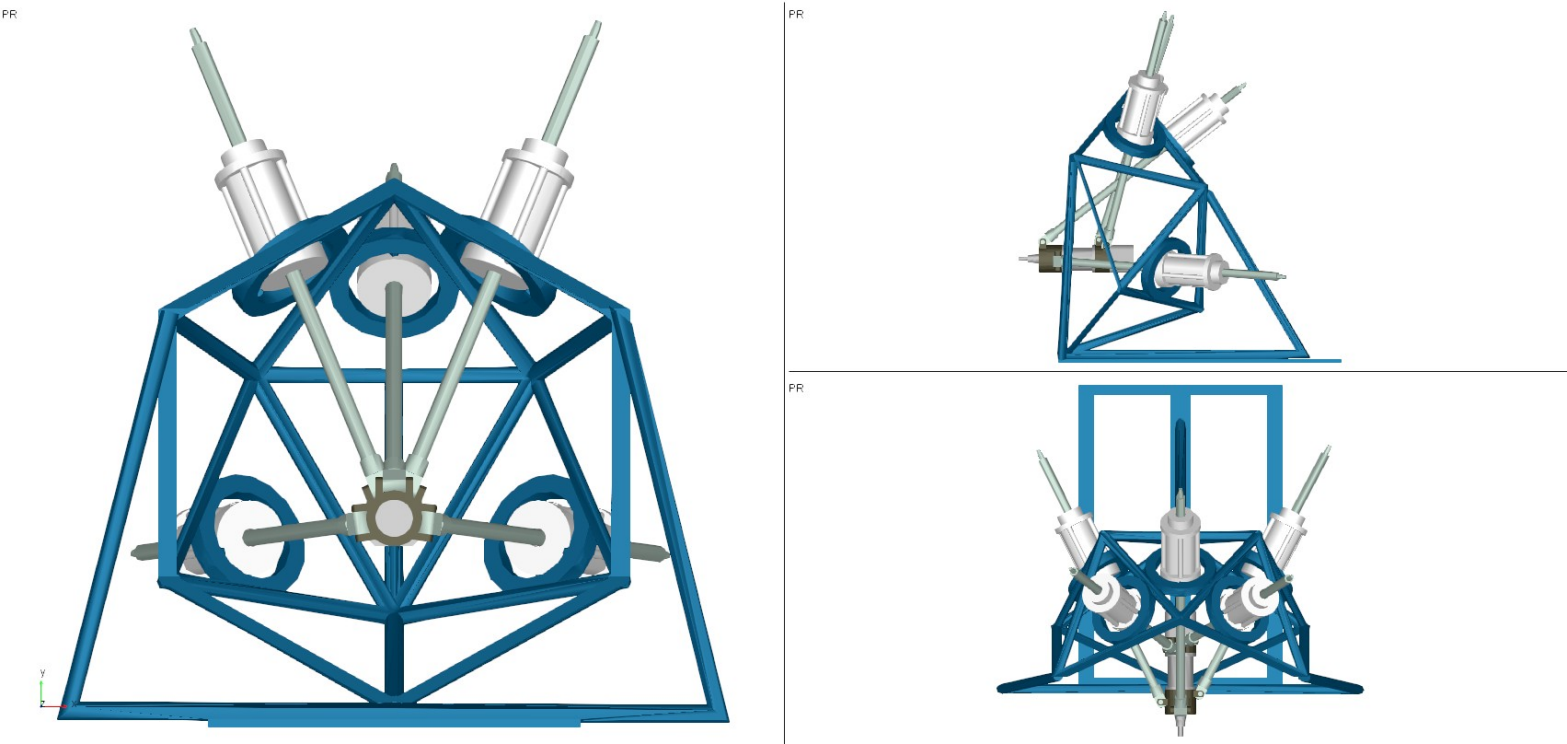
3D model of the 4SPRR-SPR parallel robot.

The ADAMS software is used for simulation to verify the effectiveness of the proposed algorithm. The algorithm results are compared with the simulation results. The 3D model of the 4SPRR-SPR parallel robot is established and is imported into ADAMS. Each motion pair is set according to the robot configuration, as shown in Fig 18.

The moving pair, which changes the length of each limb and drives the end effector, is used as the drive of the robot. Eqs (42)–(46) show the changes in drive rod length.

$$l_{1}(t)=50(1-e^{-\pi t})\cos1.88\pi t+1900.87.$$ (42)

$$l_{2}(t)=50(1-e^{-\pi t})\sin1.88\pi t+1589.06.$$ (43)

$$l_{3}(t)=50\cos t+1517.89.$$ (44)

$$l_{4}(t)=75\sin t+1413.79.$$ (45)

$$l_{5}(t)=75(1-e^{-\pi t})\sin0.86\pi t+1393.17.$$ (46)

**Fig 19:**
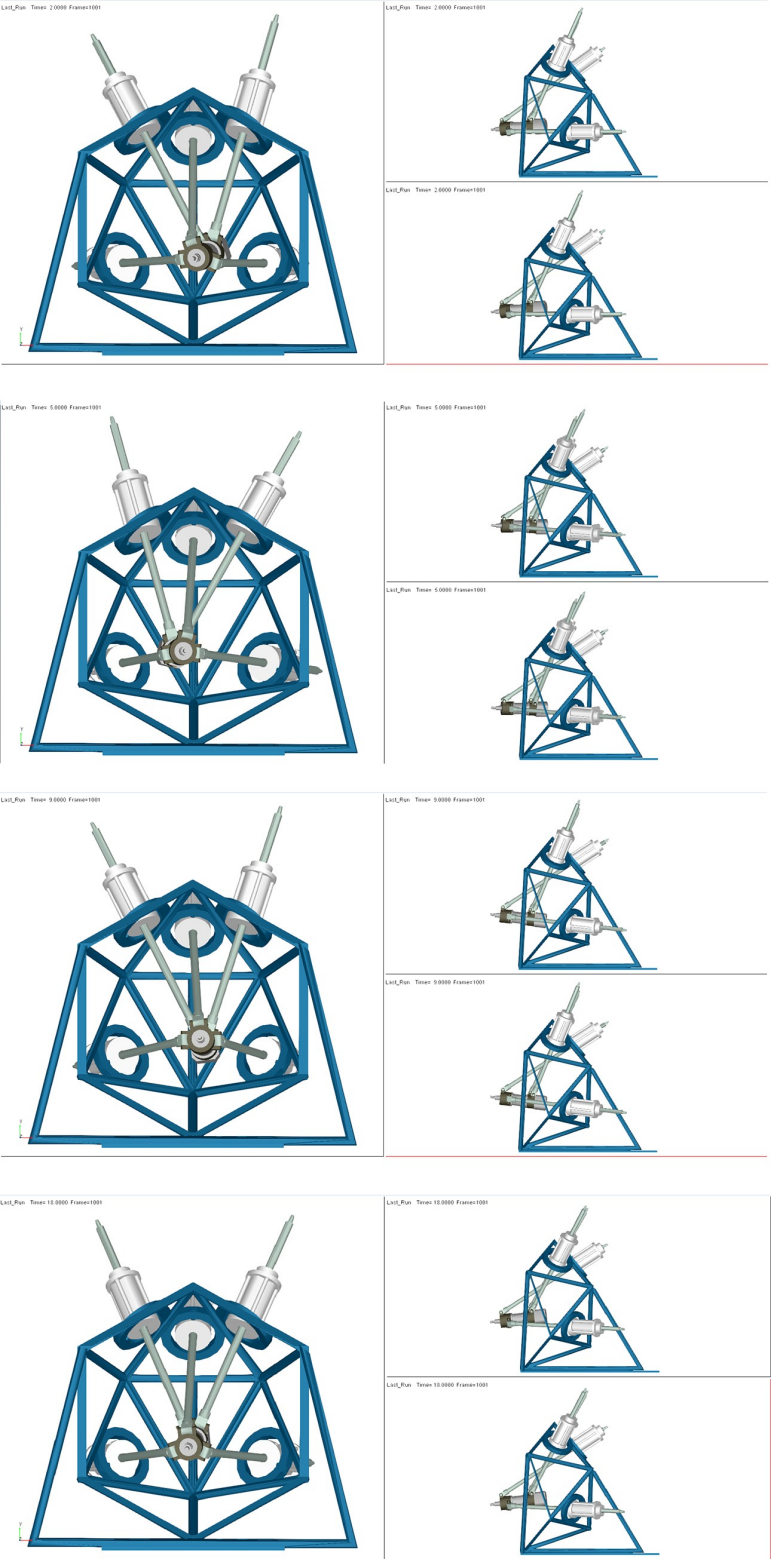
The pose of the end actuator for different values of time *t*. (A) *t* = 2. (B) *t* = 5. (C) *t* = 9. (D) *t* = 18.

The range of time t and the simulation step are set to 0 to 20 s and 0.02 s, respectively. The pose of the end actuator driven by the limbs is shown in Fig 19.

**Fig 20:**
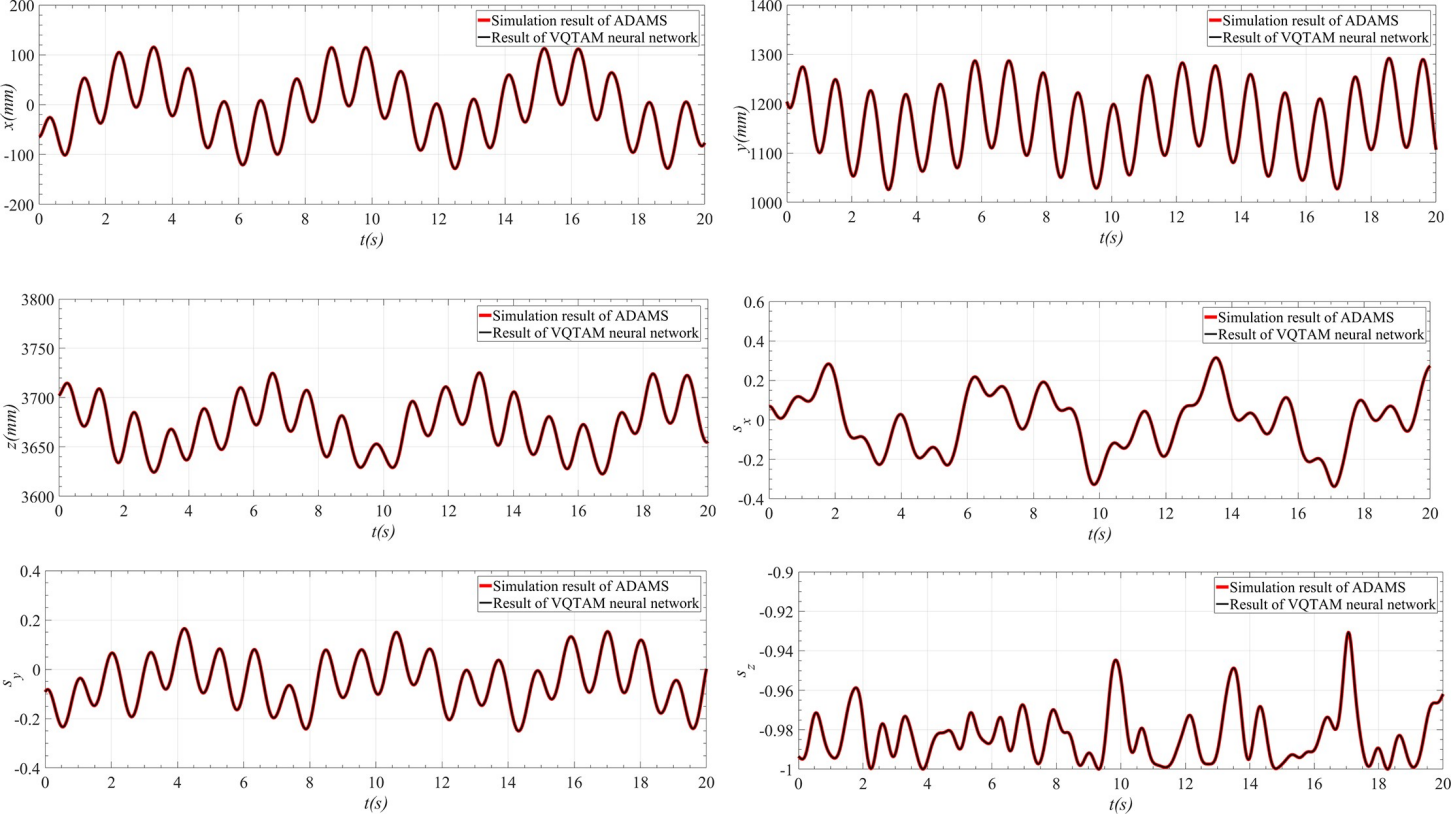
Comparison between the simulation results and VQTAM output. (A) Simulation result and VQTAM output of *x*. (B) Simulation result and VQTAM output of *y*. (C) Simulation result and VQTAM output of *z*. (D) Simulation result and VQTAM output of *s*_*x*_. (E) Simulation result and VQTAM output of *s*_*y*_. (F) Simulation result and VQTAM output of *s*_*z*_.

The forward kinematics simulation results and the poses [*x*, *y*, *z*; *s*_*x*_, *s*_*y*_, *s*_*z*_] of the parallel robot are obtained using ADAMS and are compared with the output results of the VQTAM neural network, as shown in Fig 20.

**Fig 21:**
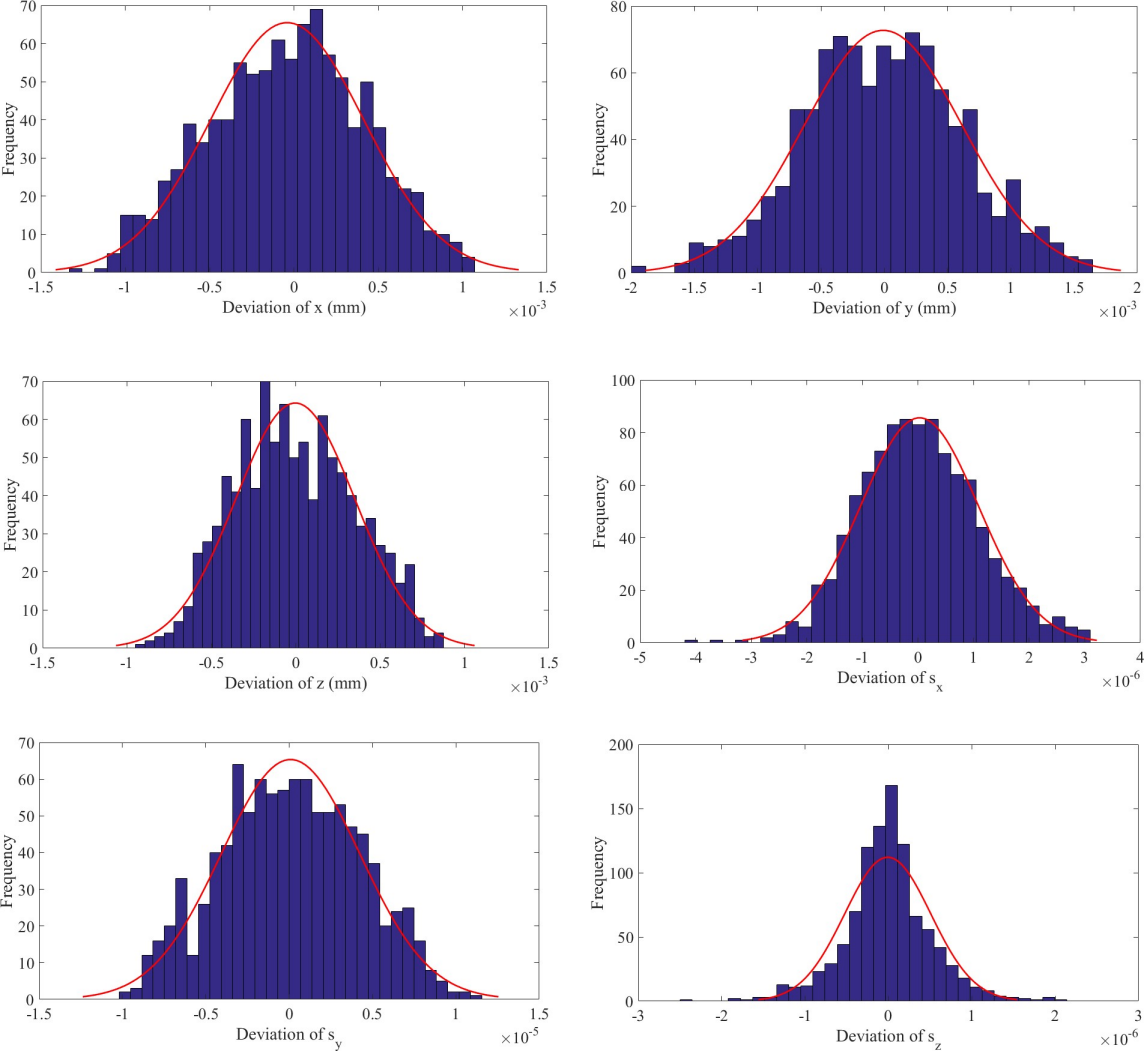
Deviation between simulation result and VQTAM output. (A) Deviation of *x*. (B) Deviation of *y*. (C) Deviation of *z*. (D) Deviation of *s*_*x*_. (E) Deviation of *s*_*y*_. (F) Deviation of *s*_*z*_.

As seen in Fig 20, the simulation results obtained using ADAMS are consistent with the output results of the VQTAM neural network, which indicates that the two forward kinematics calculation methods have the same effect. The deviation between the two results is shown in Fig 21 and Table 7.

**Table 7:** Maximum deviation between the two results.

Parameter	*x* (mm)	*y* (mm)	*z* (mm)	position deviation (mm)
Deviation	0.0013	0.0019	0.0009	0.0020
Parameter	*s*_*x*_ (×10^−6^)	*s*_*y*_ (×10^−5^)	*s*_*z*_ (×10^−6^)	attitude deviation (×10^−5^)
Deviation	4.0020	1.1325	2.4168	1.1733

The simulation results of ADAMS and the output of the VQTAM neural network have little deviation in the case of forward kinematics of the 4SPRR-SPR parallel robot. The order of magnitude of the position deviation of the two results is 10^−3^ mm, and the order of magnitude of attitude deviation is less than 10^−5^. The analysis shows that the output of the VQTAM neural network agrees with the simulation results of ADAMS. However, the calculation efficiency of ADAMS is low. By contrast, the algorithm proposed in this paper is more suitable for applications with high real-time requirements.

Table 8 shows compares the forward kinematics solution obtained using the proposed VQTAM neural network with those obtained using other methods. The comparison is made primarily on the basis of the generality, efficiency, precision, and simplicity of implementation. The intelligent algorithm is restricted by its iterative efficiency, and a corresponding objective function must be built for the optimization object, which limits the applicability of this algorithm. The performance of a neural network is strongly dependent on the initialization of hyper-parameters, which complicates the application process. As the construction of the VQTAM neural network is data-driven, the kinematics based on VQTAM is not limited to the 4SPRR-SPR parallel robot. The application of the VQTAM neural network can be extended to the forward kinematics of other parallel robots and the inverse kinematics of serial robots. The VQTAM neural network does not need other prior parameters, which simplifies its implementation.

**Table 8:** Comparison of different methods for forward kinematics of parallel robot.

	Generality	Efficiency	Precision	Simplicity in implementation
Intelligent optimization algorithm [23–28] (GA, DE, PSO, etc.)	average	low	high	low
Neural network [29–33]	average	high	high	average
NARX [34]	high	high	average	average
VQTAM neural network used in this paper	high	high	high	high

**Fig 22:**
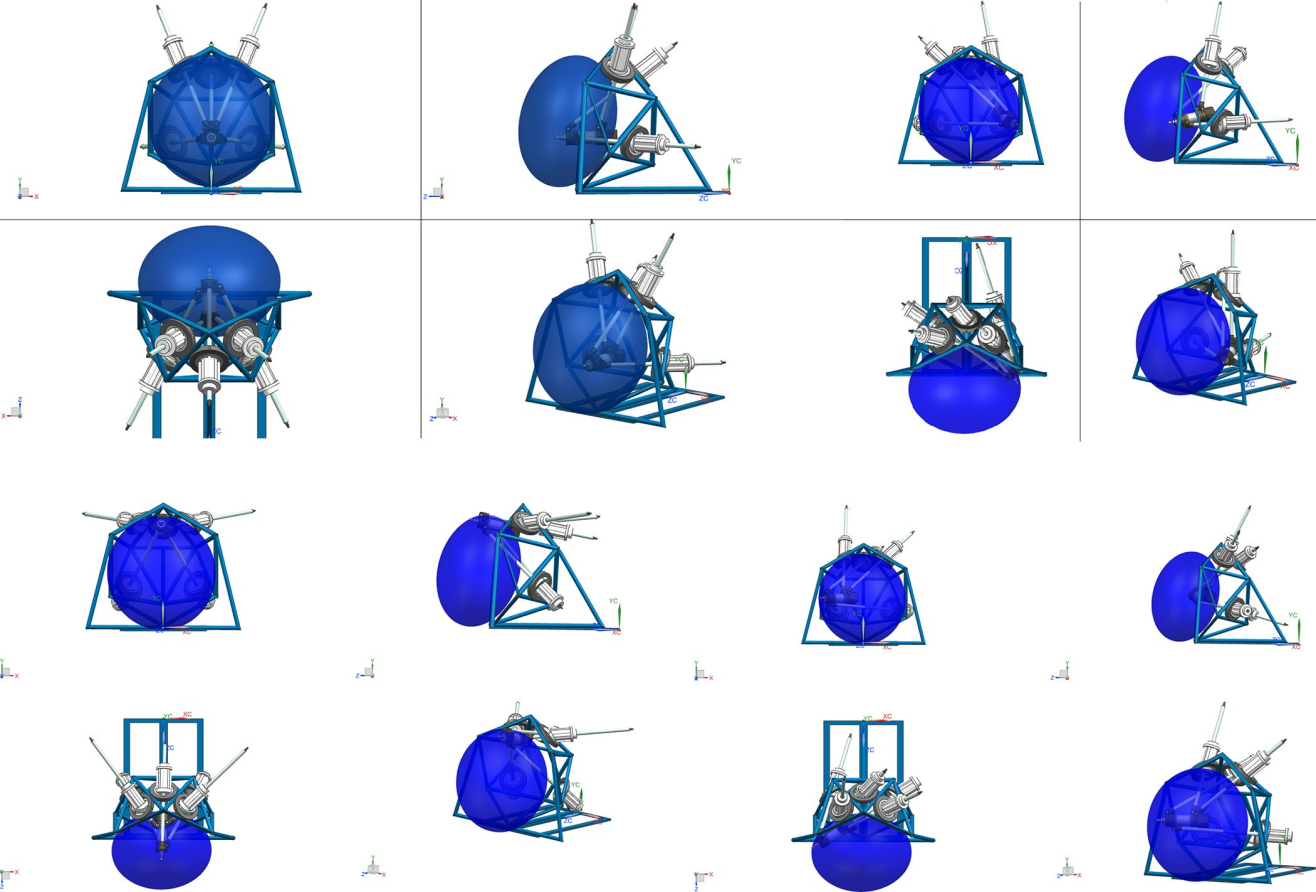
3D model and workspace. (A) The normal working state of the end actuator in the workspace. (B) The limit state 1 of the end actuator in the workspace. (C) The limit state 2 of the end actuator in the workspace. (D) The limit state 3 of the end actuator in the workspace.

As shown in Fig 22, the workspace boundary of the parallel robot calculated using the proposed boundary extraction algorithm can be imported into the 3D model to directly observe its relative position.

In Fig 22, the blue translucent ellipsoid represents the workspace boundary of the parallel robot. In Fig 22A, the length of each driving rod is normal when the reference point of the robot end actuator is in the workspace. Fig 22B–22D show that at least one driving rod reaches the limit when the reference point of the robot end actuator is located on the boundary of the workspace. The above analysis shows that the proposed algorithm produces effective results, and the fitted ellipsoid can also effectively describe the size and location of the workspace.

Table 9 shows the comparison of the workspace extraction algorithm proposed in this paper with those used in other methods for workspace analysis. Most existing workspace analysis methods are applicable for a specific robot configuration and are highly effective for the corresponding robot. However, they cannot be extended to other robot configurations. The proposed workspace analysis method based on topological geometry has advantages with reference to generality, efficiency, and accuracy.

**Table 9:** Comparison of different methods for workspace analysis of parallel robot.

	Generality	Efficiency	Precision	Simplicity in implementation
Global search algorithm [19–22]	high	low	high	high
Boundary extraction algorithm in ref. [40, 41]	low	average	high	average
Monte Carlo Method [42, 43]	low	average	high	average
Boundary extraction algorithm described in this paper	high	high	high	average

## 7. Conclusion

The geometric characteristics of the 4SPRR-SPR parallel robot are analyzed based on its kinematic pair configuration, and the inverse kinematic solution algorithm of the robot is deduced. The rod length variation obtained from the inverse solution of the parallel position is differentiated, and the velocity and acceleration of each rod in the process of mechanism action are obtained.

A fast forward kinematics mapping method for parallel robots using VQTAM for nonlinear dynamic systems identification is proposed. To further optimize the prediction accuracy of the network and reduce the dimension of the network in application, an improved LLE algorithm for VQTAM is proposed. The VQTAM algorithm and its improved version are tested. The estimation accuracy can be optimized for a low-dimensional network using the VQTAM local linearization improvement algorithm. The above algorithm can show good prediction accuracy. The kinematics analysis of a 4SPRR-SPR parallel robot provides a basis for kinematics and dynamics analysis as well as a theoretical basis for trajectory planning and attitude control of the mechanism. The kinematics based on VQTAM is not only limited to the 4SPRR-SPR parallel robot, but the application of the VQTAM neural network can also be extended to the forward kinematics of other parallel robots and inverse kinematics of serial robots.

A boundary extraction algorithm for the workspace analysis of the mechanism is proposed based on topological geometry. The time complexity is analyzed and compared with that of traditional algorithms. The boundary extraction algorithm has higher efficiency and lower dependence on initial search conditions compared to the global search algorithm. The workspace of the 4SPRR-SPR parallel robot is solved using the boundary search algorithm. The validity of the algorithm is verified, which provides support for the workspace optimization of the robot. The workspace analysis method based on topological geometry proposed in this paper has advantages with reference to generality, efficiency, and accuracy.

A 3D model of the 4SPRR-SPR robot is established, and a kinematics simulation is performed using ADAMS. The simulation results are compared with the output results of the VQTAM neural network, and the order of the magnitude of deviation is found to be within 10^−3^. The validity and accuracy of the kinematic solution obtained using the VQTAM neural network are verified. The workspace obtained using the boundary extraction algorithm is validated using the 3D model of the parallel robot, which demonstrates the reliability of the workspace analysis method proposed in this paper.

## Supporting information

S1 Table Test data for inverse kinematics algorithm of parallel robot. (XLSX)

S2 Table Test data for VQTAM network. (XLSX)

S3 Table Test data for workspace analysis of Stewart platform. (XLSX)

S4 Table Test data for the workspace analysis of the 4SPRR-SPR parallel robot. (XLSX)

S5 Table Test data for comparison between VQTAM and simulation results. (XLSX)
